# Self‐Nitriding Nanostructured Transition Metal Nitrides in Architected‐Carbon Matrices: Unveiling Mechanisms and Advancing Performance in Lithium‐Sulfur Pouch Cells

**DOI:** 10.1002/advs.202521940

**Published:** 2026-03-31

**Authors:** Yael Rodriguez Ayllon, Liqiang Lu, Dongjiu Xie, Xuefeng Pan, Teodor Jianu, Fangmu Qu, Sijia Cao, Yu Zhang, Roberto Félix, Marcus Bär, Nadezda V. Tarakina, Johannes Schmidt, Qingping Wu, Jiayin Yuan, Yan Lu

**Affiliations:** ^1^ Institute of Electrochemical Energy Storage Helmholtz‐Zentrum Berlin für Materialien und Energie Berlin Germany; ^2^ Institute for Technical and Environmental Chemistry Friedrich‐Schiller‐Universität Jena Jena Germany; ^3^ Department of Colloid Chemistry Max Planck Institute of Colloids and Interfaces Potsdam Germany; ^4^ Department of Chemistry Stockholm University Stockholm Sweden; ^5^ Department of Interface Design Helmholtz‐Zentrum Berlin für Materialien und Energie Berlin Germany; ^6^ Energy Materials In‐Situ Laboratory Berlin (EMIL) Helmholtz‐Zentrum Berlin für Materialien Und Energie Berlin Germany; ^7^ Department of X‐ray Spectroscopy at Interfaces of Thin Films Helmholtz Institute Erlangen‐Nürnberg For Renewable Energy (HIERN) Berlin Germany; ^8^ Department of Chemistry and Pharmacy Friedrich‐Alexander‐Universität Erlangen‐Nürnberg (FAU) Erlangen Germany; ^9^ Department of Chemistry Functional Materials Technical University Berlin Berlin Germany; ^10^ Chongqing Institute of Green and Intelligent Technology Chinese Academy of Sciences Chongqing China; ^11^ Helmholtz Institute For Polymers in Energy Applications (HIPOLE Jena) Jena Germany

**Keywords:** Li‐S batteries, nanostructured transition metal nitrides, Poly(ionic liquid), pouch cells, reaction kinetics

## Abstract

Transition metal nitrides (TMNs) are attractive for cutting‐edge energy storage technology, especially emerging lithium–sulfur (Li–S) batteries, owing to their electronic structures resembling those of noble metals. Herein, we unveil the underlying mechanism by which TMNs accelerate reaction kinetics, showcasing two nanostructured TMNs (Mo_2_N and VN) embedded within tailored carbon architectures. A novel, unexplored self‐nitriding approach was developed to synthesize TMNs with precisely controlled solid (sC) or hollow (hC) carbon architectures, achieved through a colloidal route using imidazolium‐based poly(ionic liquid) (PIL) nanoparticles as both a nitrogen‐rich template and morphology‐directing agent. Compact TMN architectures as sulfur hosts enhance ion diffusion and reaction kinetics, enabling efficient active site access and delivering high performance, such as VN@sC with high initial capacity of 792 mAh g^−1^ at 2 C and cyclability up to 650 cycles. Meanwhile, hollow architectures (VN@hC and Mo_2_N@hC) featuring hierarchical porous structures serve as cathode electrocatalytic additives, enabling high sulfur loading and delivering an initial capacity of 1143 mAh g^−1^ at 0.1 C. Remarkably, this performance is achieved with only 5 wt% additive content in scalable 7.9 × 11 cm^2^ and 12‐layer pouch cells designed for drone power systems.

## Graphical abstract

Imidazolium PIL nanoparticles, as an N‐rich template and morphology director, enable a colloidal self‐nitriding route to TMNs (VN, Mo_2_N) within controlled solid/hollow carbon architectures. As sulfur hosts/additives, TMNs accelerate Li–S redox kinetics and support high‐loading cathodes. This safe, green, scalable process lowers costs and enables multilayer pouch cells to deliver high energy density of 233 Wh kg^−1^ at E/S 3.4 µL mg^−1^, sufficient to power drones.

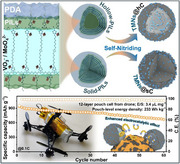

## 1 Introduction

Lithium–sulfur (Li–S) batteries stand out as highly promising candidates to drive the concept of the new generation of practical batteries. This is primarily due to their exceptional characteristics, including remarkably high theoretical specific capacity and energy density [1, 2, 3]. Although the Li‐S batteries hold attractive promise for commercialization, it has also been accompanied by several challenges mainly attributed to the intrinsic chemical nature of sulfur. These challenges include conductivity issues, volume expansion, and the manifestation of the “shuttle effect” caused by soluble intermediate lithium polysulfides (LiPSs) formed during the charge‐discharge process, thereby hindering their effective performance [2, 4, 5].

To address these challenges, various carbon‐based nanomaterials have been used as cathode hosts that can serve as both a container and a reactor for sulfur and its conversion reactions [6, 7]. However, the physical confinement of sulfur provided by the non‐polar carbon is generally insufficient to efficiently retain soluble LiPSs [8, 9, 10, 11]. An alternative strategy has been incorporated, involving the chemical anchoring of LiPSs using nanostructured polar transition metal compounds [4, 12, 13, 14, 15]. Among various metal compounds, nanostructured transition metal nitrides (TMNs) have emerged as a robust material with outstanding electrical conductivity, positioning them as a great promise in addressing the challenges [16, 17]. Recently, TMN‐based materials have been also studied as efficient electrocatalysts for sulfur redox reactions, significantly boosting overall kinetics due to their exceptional structural and electronic stability [18]. This brings tailored TMNs as cutting‐edge materials for advanced sulfur mediators in Li–S cells [19, 20, 21]. For instance, vanadium nitride (VN) nanoribbon and graphene composites not only offer a strong anchoring effect for LiPSs but also exhibit lower polarization compared to graphene alone, thanks to the high conductivity of VN [22]. This promotes faster redox reaction kinetics, leading to enhanced rate capability and cycling performance.

Porous carbon materials with high specific surface area and various spatial structures are considered to be ideal supports for TMNs (e.g., VN‐loaded hollow carbon spheres [23], MoN‐decorated carbon nano‐octahedron [24, 25], Co_3_Mo_3_N‐loaded bead‐on‐string carbon [26]) to create more efficient electrocatalytic conditions to further improve the performance of Li–S batteries. These 3D matrices facilitate the uniform distribution of sulfur within the catalytic host, ensuring high utilization of active materials and allowing for unobstructed diffusion of LiPSs intermediates. In addition, to obtain Li–S batteries with high energy density, it is required higher sulfur loading and lean electrolyte conditions [27, 28]. However, suboptimal hollow structures (or multilayer structures) might not only cause a large accumulation of redeposited sulfur, but also provide more electrochemical reaction interfaces even for parasitic reaction, leading to increased electrolyte consumption. In addition, the tortuous pathways for Li ions/electrons transport are not conducive to weakening polarization overpotential, further affecting the overall performance of battery [29]. Manipulating the spatial configuration of carbon matrix loaded with TMNs to balance the completeness (i.e., specific capacity) and kinetics (i.e., rate performance) of LiPSs conversion reactions toward high‐energy‐density Li–S, remains a formidable challenge.

Furthermore, the manufacturing of nanostructured TMNs (mainly ammonia‐based reduction) faces challenges in terms of their universality, safety concerns and scalability, indicating the need for progression in this field [30]. Conversely, poly(ionic liquid)s (PILs) represent a distinct class of functional polyelectrolytes, distinguished by the incorporation of ionic liquid (IL)‐type moieties within each repeating unit [31, 32, 33]. Imidazolium‐based PILs, where the imidazolium group is incorporated into each repeating unit, have garnered significant attention, being the most extensively studied PILs. Their imidazolium functionality imparts highly tunable physicochemical properties, influenced by the choice of counteranion, IL monomer concentration, and the balance between hydrophobic and hydrophilic domains [32, 33, 34]. Moreover, the synthesis of PILs via a facile polymerization‐induced self‐assembly (PISA) approach provides precise control over the desired polymer morphology, enabling a reliable formation of well‐defined nanostructures. These nanostructures could serve as sacrificial templates, effectively preserving their initial morphology during subsequent heat treatment. Multiple studies have provided evidence of the synergistic effect of nitrogen‐rich PILs as high‐quality nitrogen‐doped carbon precursors [35]. In our previous study, we have demonstrated that organo‐metallic ion complexes based on PILs have emerged as promising soft templates for the synthesis of ultrafine and homogeneous metal‐based nanoparticles through direct reductive calcination [36]. These complexes, formed by coordinating metal ions with the functional groups present in PILs, provide a controlled environment for nucleation and growth of the nanoparticles [36, 37, 38]. However, the multistep synthesis procedures, including the requirement for an additional nitrogen source, still present significant challenges for practical scalability.

In this work, we explored the mechanism of nanostructured TMNs in catalytic environments for LiPSs conversion kinetics, leveraging the advantages of imidazolium‐based PILs to effectively design varied spatial configurations of TMNs. The synergistic effect of imidazolium‐type PILs in the construction of various TMNs without external nitridation process was first unveiled through a straightforward approach assisted by a coating solution made of different transition metal salts and dopamine hydrochloride (DA). The electrochemical properties of VN and Mo_2_N nanoparticles embedded in solid and hollow carbon matrices were systematically compared, evaluating both their affinity for LiPSs and their catalytic activity. Vanadium nitride embedded in solid carbon spheres (VN@sC) exhibited superior LiPSs conversion kinetics for Li–S coin cells, likely due to the unique confined structure combined with the high content and uniform distribution of VN nanoparticles, which facilitate shorter transport distances for Li ions and electrons alongside moderate adsorption capacity. Additionally, the configuration promotes more efficient electrochemical reaction interfaces, reducing excessive electrolyte consumption while maintaining high sulfur utilization. Therefore, VN@sC as sulfur host has optimal cycle stability at 2 C showing an initial specific capacity of 760 mAh g^−1^, which after around 650 cycles delivered 495 mAh g^−1^ at coin cell level. On the other hand, a trade‐off effect was observed in the hollow architectures, which facilitated scalability to pouch cell applications as electrocatalytic additives. This effect is likely due to the optimized balance between nanoparticle distribution and spatial configuration, allowing for a reduction in electrocatalytic additive content up to 5% while still maintaining high sulfur utilization of 950 and 980 mAh g^−1^ for VN@hC‐ and Mo_2_N@hC‐based pouch cell after 50 cycles at 0.1 C, respectively.

## 2 Results and Discussion

**SCHEME 1:**
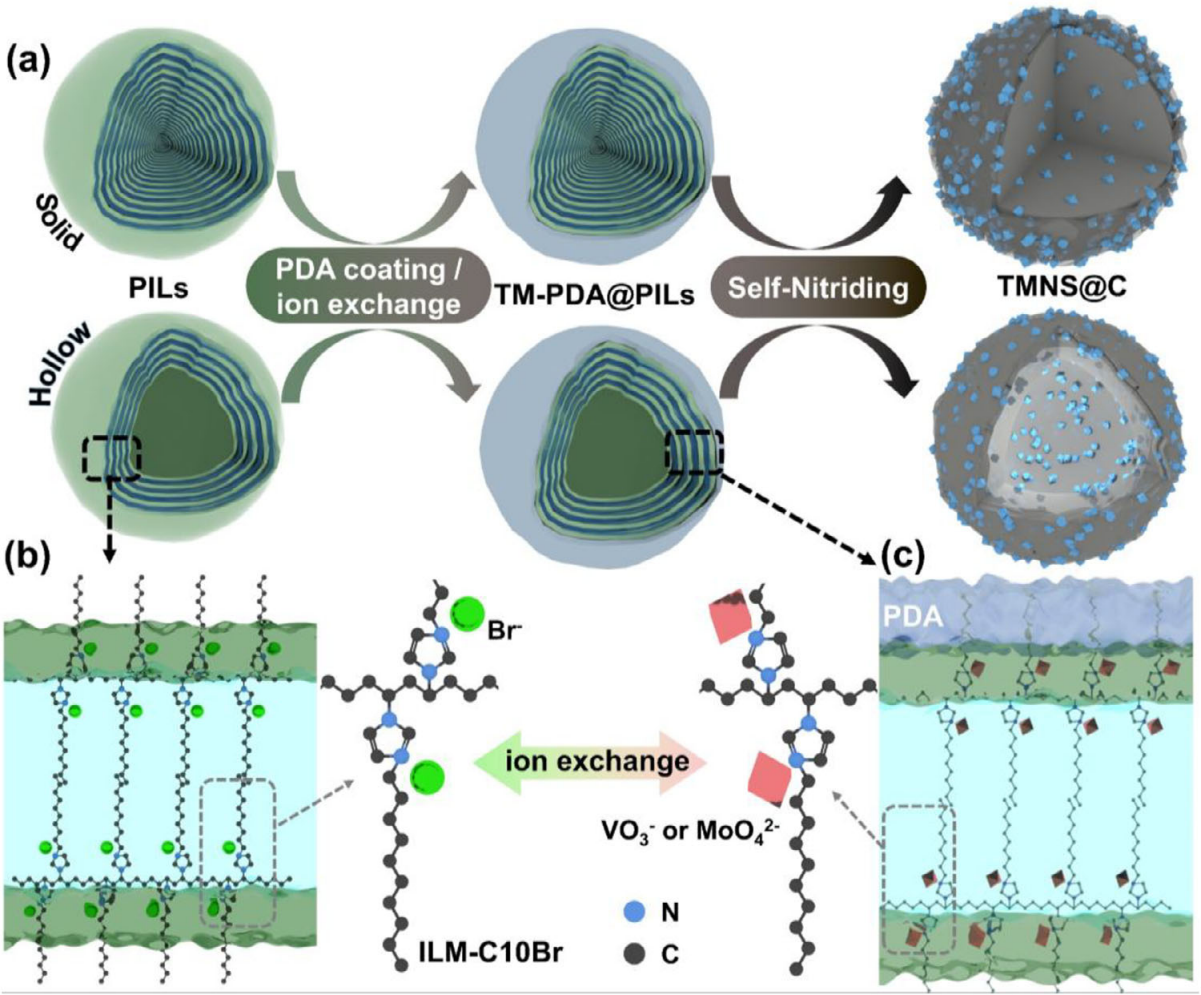
Representation of the synthesis pathway of TMNs embedded in hollow and solid carbon architectures (a). Representation of the chain assembly containing long alkyl chains and counterions attached to the imidazolium‐containing ring (b). In situ‐simultaneous ion exchange‐PDA coating (c).

We employed a strategic synthesis of nitrogen‐doped carbon substrates with customizable hollow or solid frameworks. These tailored carbon architectures were created with tunable, in situ‐loaded transition metal nitrides (TMNs), using imidazolium‐based PIL sphere as a soft template, as illustrated in Scheme 1. Overall, imidazolium‐type PILs with long alkyl substituents on the cations are marvelous amphiphilic‐like soft matter. Their synthesis was extensively described in previous work. [36, 37] PIL spheres were synthesized via polymerization of an ionic liquid monomer, 3‐*n*‐decyl‐1‐vinylimidazolium bromide (ILM‐C10Br). The homopolymerization was initiated using VA‐86 as non‐ionic initiator in water at 75°C for 20 h. After removing impurities through dialysis against DI‐water, a stable white colloidal dispersion was obtained. This stability could be achieved because of the charged hydrophilic outer layer of the spheres, formed by polymerization‐induced self‐assembly due to inherent incompatibility between the hydrophilic charged imidazolium main chain and the hydrophobic long alkyl substituents. Interestingly, the morphology of these amphiphilic PIL spheres strongly depends on the initial IL monomer concentration for the polymerization, therefore, can be easily regulated [37].

**FIGURE 1:**
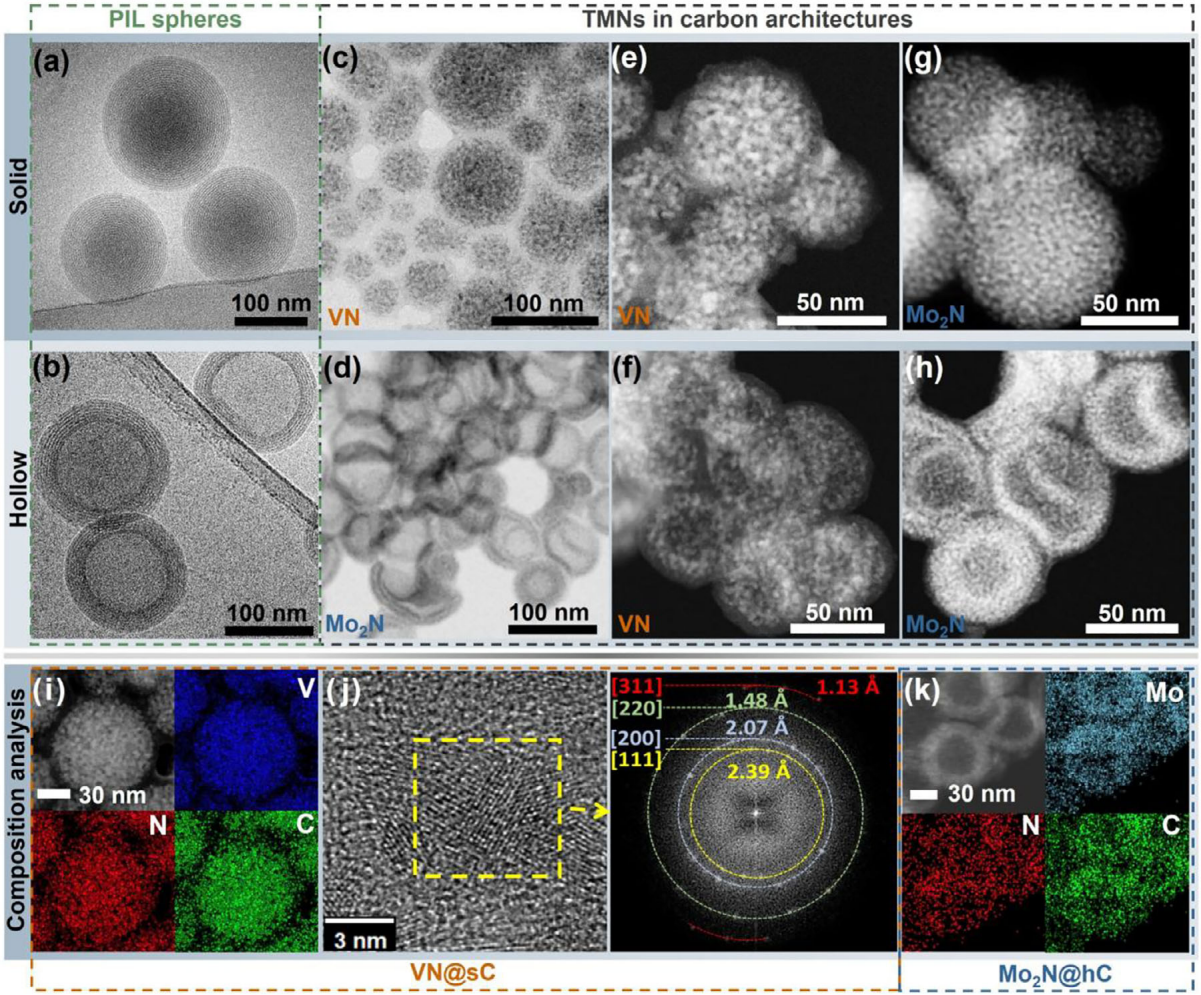
Cryo‐TEM images of solid‐dense onion‐like PIL spheres (a), and hollow PIL spheres (b). BF‐STEM overview of VN@sC (c), and Mo_2_N@hC (d), ADF‐STEM of VN@sC (e), VN@hC (f), Mo_2_N@sC (g), and Mo_2_N@hC (h). EDX mapping of VN@sC (i), HRTEM of a randomly selected spot of VN@sC and its FFT pattern (j), and EDX mapping of Mo_2_N@hC (k).

The drying effect on conventional transmissions electron microscopy (TEM) hinders the native structure of PIL particles (e.g. electron density, packaging and distributions of ions), as depicted in Figure. S1. Therefore, cryogenic TEM (cryo‐TEM) was applied to verify the preserved inner structure of the PIL spheres. As shown in Figure 1a, solid, densely packed onion‐like spheres were formed when the ionic liquid (IL) monomer was polymerized at a concentration of 24 g L^−1^. In contrast, reducing the IL monomer concentration to 12 g L^−1^ resulted in the formation of hollow spheres, as illustrated in Figure 1b. Despite their distinct overall spherical morphologies, both PIL architectures display repeated patterns of centric lamellas, alternating layers within the nanoscale domain. This contrast can be attributed to the higher electron density of the bromide anions on the charge backbone in contrast to the alkyl chains [32, 37].

Due to the fascinating features of such imidazolium‐based PIL spheres, the modulation of the counterion attached to the nitrogen‐containing ring may play a crucial role in design of hybrid materials (Scheme 1b). In this case, anion exchange is an effective, straightforward strategy to introduce metal ionic species into PILs. When both solid‐dense and hollow PIL spheres underwent direct anion exchange with inorganic salts (NH_4_VO_3_ or (NH_4_)_2_MoO_4_), a flocculation phenomenon was observed, as depicted in Figure S2. This could be attributed to both the size and the valence of the replacing counterions, which cause a rapid cross‐linking of the PIL polymer chains. Such severe aggregation could lead to the uncontrollable crystal growth of metal species during subsequent pyrolysis [35, 39, 40]. To overcome these issues, polydopamine (PDA) has been introduced as a protective thin layer [41, 42], which could not only enhance the colloidal stability of the PIL particles, but also provide a controlled ionic release to the PILs. The coordination of several transition metals to moieties containing catechol groups has been widely reported [43, 44, 45]. During the polymerization of dopamine, the catechol groups, which initially chelate metal ions, are oxidized to quinones [41, 42]. As the polydopamine (PDA) layer forms on the surface of PIL spheres, these chelated metal ions may be released and exchanged into the PIL matrix due to changes in the coordination environment or competitive binding interactions, resulting in a metal‐functionalized‐PIL particle covered by a thin layer of PDA (TM‐PDA@PILs) (Scheme 1c). In Figure S2, the deep‐black colloidal dispersion of TM‐PDA@PILs after ionic exchange‐assisted PDA coating can be observed, which shows enhanced colloidal stability without direct flocculation. Moreover, TEM provided an overview of the TM‐PDA@PIL particulate morphology after the reaction (Figure S3). The characteristic lamellae pattern is observed evenly in all systems containing V and Mo ions, suggesting that the original morphology can be maintained after effective ionic exchange. This straightforward one‐pot method appears to tackle the flocculation issue effectively, while controlling the ionic exchange process.

TMNs@C spheres are constructed by a direct thermal decomposition of the as‐obtained TM‐PDA@PILs (TM = Mo or V) in argon atmosphere, with the nitrogen source derived exclusively from the self‐nitriding PILs template. The fabrication of nanostructured Mo_2_N@C and VN@C with both hollow (denoted as TMNs@hC) and solid (denoted as TMNs@sC) morphologies was investigated. These materials were synthesized at the minimum temperatures required for nitride phase formation (600°C for Mo_2_N and 700°C for VN) and characterized using conventional TEM. Figure S4 reveals the overall TMNs@C morphologies. It was found that, apart from the initial template morphology, there is no major difference in the final architecture of TMN@C synthesized under these conditions, indicating a relatively minor influence of the TMN source. For the Mo_2_N@sC and the VN@sC (Figure S4a,b), well‐defined spheres are observed after thermal treatment, showing uniformly dot‐like nanoparticles embedded within the amorphous carbon matrix. Figure S4c,d displays the Mo_2_N@hC and VN@hC structures, which consist of hollow carbon spheres with a distorted, erythrocyte‐like shape, decorated with dot‐like nanoparticles. To date, a variety of nitridation strategies have been reported. Organic‐based nitridation routes typically produce additional carbonaceous residues that require post‐synthesis washing or disposal, whereas gas‐phase NH_3_ nitridation mainly introduces safety concerns while generating minimal solid waste. The herein introduced TMN synthesis route involves fewer processing steps, lower nitridation temperatures, ammonia‐free conditions, and minimal solid waste generation, offering improved scalability at reduced cost. Table S1 summarizes and compares these reported syntheses protocols with the approach presented in this work.

Scanning transmission electron microscopy (STEM) provides a consistent analysis on the TMN crystallites distributed in the carbon matrix with very high spatial resolution. As shown in Figure 1c,d, the Bright‐Field STEM (BF‐STEM) overview images of VN@sC and Mo_2_N@hC reveal solid and hollow spherical morphologies consistent with those of their precursor materials, while the obtained carbon matrix is amorphous. The darker contrast regions are ascribed to TMN assemblies. Figure S5a–d presents the Bright Field (BF‐STEM) images for all the systems studied. To further investigate the spatial distribution of transition metals within the matrix, annular dark‐field (ADF) STEM imaging was performed (Figure 1e–h). The bright contrast features correspond to TMN domains, owing to the higher scattering intensity of metal atoms relative to carbon. The solid spheres exhibit a uniform dispersion of VN or Mo_2_N throughout the carbon framework, whereas the hollow spheres display evenly distributed bright spots along the carbon ring. Moreover, energy‐dispersive X‐ray spectroscopy (EDS) provides an overall mapping of elements within VN@sC and Mo_2_N@hC, as shown in Figure 1i,k, confirming that all elements are homogeneously distributed throughout the entire carbon matrix. Figure 1j depicts the High‐resolution TEM (HRTEM) images, together with their corresponding Fast Fourier Transform (FFT) patterns, reveal bright spots in the FFT indicative of the crystalline nature of the TMNs, while the diffuse halo arises from the amorphous carbon matrix, which aligns well with the structural contrast observed in both BF and ADF STEM images. The lattice fringes of the crystal domains corresponding to VN revealed nanodots with approximately 3 nm in size. The interplanar distances calculated from FFT are correlated with specific Miller indices, measuring 0.239, 0.207, 0.148, and 0.113 nm, which can be ascribed to the (111), (200), (220), and (311) planes, respectively. The slight deviation from the values reported in the literature (VN PDF#35‐0768), particularly for the (200) and (311) planes can be attributed to oxygen contamination, which likely stems from the surface passivation occurring post‐synthesis. A similar phenomenon was consistently observed across the remaining samples, as illustrated in Figure S6, where similar deviations were noted for both TMNs. Furthermore, electron energy loss spectroscopy (EELS) provides insights into the chemical structure by analyzing the core‐loss edges: V‐L_2,3_ and N‐K for VN, and Mo‐M_2,3_ for Mo_2_N. Figure S7 presents the electron energy loss spectra for Mo in Mo_2_N, embedded in both solid and hollow carbon, as well as V and N in VN. The Mo‐M_2_ edge for both Mo_2_N@hC and Mo_2_N@sC was observed around 411.9 eV, while the Mo‐M_3_ edge appeared at approximately 394.6 eV. It is noted that the N‐K edge could not be quantified due to overlapping with Mo edges themselves. On the other hand, for VN samples, the V‐L_3_ edge was observed at around 515.1 eV and the V‐L_2_ edge around 521.6 eV. The N‐K edge displayed a doublet at around 398.3 and 408 eV, which is characteristic for VN [46]. The presence of the specific core‐loss edges corresponding to Mo_2_N and VN confirms the bonding between molybdenum or vanadium and nitrogen at the core level.

By understanding the relationship between the thermal treatment temperature and the crystallite size of TMNs, insights can be gained into the formation mechanisms and structural evolution of TMNs@C spheres. In this analysis, the formation of TMNs@sC from TM‐PDA@PILs was investigated under varying thermal treatment conditions, ranging from the minimal temperature required to form the nitride phase to 1000°C. TEM images of the Mo_2_N@sC prepared at 600°C, 800°C, and 1000°C are introduced in Figure S8a–c, meanwhile the VN@sC prepared at 700°C, 800°C, and 1000°C are introduced in Figure S8d–f. It is noted that from the minimum TMN formation temperature, well‐distributed carbon spheres resembling sesame‐ball‐like structures decorated by Mo_2_N or VN nanoparticles, were obtained, as meticulously observed in the insert of the image. As the temperature increased to 800°C, it was observed that the Mo_2_N and VN nanoparticles within the carbon sphere matrix became larger and sharper compared to those at the initial formation temperature. When the temperature was further raised to 1000°C, a clear morphological change occurred, with the structures resembling aggregated metal clusters encapsulated within carbon spheres rather than simply decorated spheres. This indicates not only crystallite growth but also a redistribution of the crystallite phase throughout the carbon matrix, with a notably larger crystallite size.

Thermogravimetric analysis coupled with mass spectrometry (TGA‐MS) provides direct chemical evidence for the self‐nitriding process (Figures S9 and S10). For V‐PDA@PILs heated in Ar, NH_3_ (m/z 17) starts to evolve at about 190°C and follows the main mass loss up to roughly 400°C, showing that the nitrogen‐rich PIL acts as an internal nitrogen reservoir. At higher temperatures the dominant nitrogen‐containing product is N_2_ (m/z 28), accompanied by a parallel fragmented N signal (m/z 14), which peaks between about 350°C and 500°C and coincides with VN formation. CO_2_ and NO_2_ increase during carbonization and the O_2_ signal decreases, consistent with oxygen removal. Together with a residual yield of about 30 wt% at 800°C, these features demonstrate that nitrogen required for metal nitridation is supplied by thermal decomposition of the PIL framework itself, thus validating the proposed self‐nitriding pathway without using any external nitrogen source. Nitrogen utilization is defined as the fraction of nitrogen from the precursor retained in TMN after thermal treatment, relative to the nitrogen content of the precursor. The PIL precursor has a nitrogen content of 8.11 wt%, as confirmed from the elemental analysis shown in Table S3. Based on the measured nitrogen content, the nitrogen utilization is approximately 62.3% for VN@hC and 80.5% for VN@sC. For Mo_2_N@hC and Mo_2_N@sC, the utilization is approximately 50.2% and 51.1%, respectively. These results indicate the fraction of nitrogen originally present in the polymer was retained in the final transition metal nitride (TMN) products.

**FIGURE 2:**
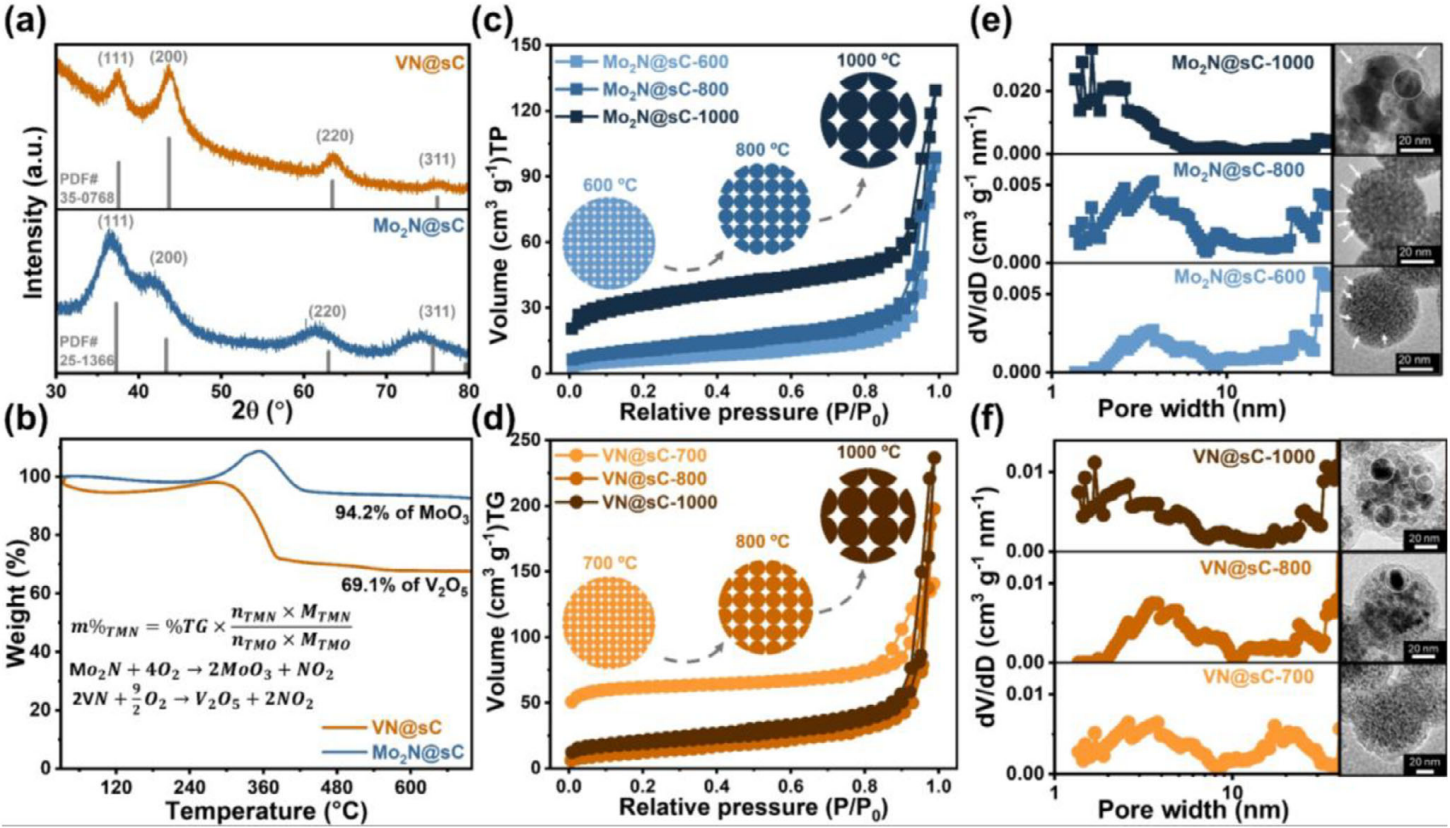
XRD patterns of Mo_2_N@sC and VN@sC (a). TGA curves of Mo_2_N@sC and VN@sC obtained in synthetic air (b). N_2_ adsorption‐desorption isotherms for Mo_2_N@sC acquired at different temperatures (c) and VN@sC (d), along with their corresponding pore size distribution and TEM images of Mo_2_N@sC (e) and VN@sC (f), respectively.

XRD analysis was conducted to further identify the crystalline phases present in the as‐synthesized TMNs embedded in hollow and solid spheres (Figure 2a and Figure S11a). XRD pattern of Mo_2_N embedded in both hollow and solid carbon spheres displayed several broad peaks at 36.8°, 41.7°, 61.8°, and 74.5°, which can be referred to the (111), (200), (220), and (311) Miller indices (hkl planes) of Mo_2_N cubic Pm‐3m space group (Mo_2_N PDF#25‐1366), respectively. On the other hand, XRD pattern of VN embedded in both hollow and solid carbon spheres revealed several broad peaks at 37.6°, 43.9°, 63.8°, and 76.5°, corresponding to the (111), (200), (220), and (311) hkl planes of VN cubic Fm‐3m space group (VN PDF#35‐0768), respectively. The XRD patterns corresponding to TMNs@sC prepared under different annealing temperatures are included in Figure S11b,c, which are in good agreement with the TEM result and reveal an increase in peak intensity as the temperature rises. However, it is noted that the molybdenum carbide (Mo_2_C PDF#35‐0787) or vanadium carbide (V_8_C_7_ PDF#35‐0786) phases could be formed at relatively elevated temperatures and exhibited a pattern very similar to that of the TMN phase [47], which complicates characterization via XRD. Additionally, the Mo‐containing sample treated at 1000°C showed a small peak at ca. 34.5° and an irregular shoulder shifting peak between 36°–42°, which can be ascribed to (100) and (101) planes of β‐Mo_2_C. Hence, the primary factor influencing crystal growth and its chemical transformation is the temperature at which the process occurs.

TGA was conducted in synthetic air to stoichiometrically determine the TMN content. Figure 2b shows the thermal oxidation of Mo_2_N and VN embedded in solid carbon spheres. After heating to 700°C, a well‐defined constant curve was observed, indicating the full conversion. An identical experiment was conducted for TMNs@hC, with the results reported in Figure S12a. The final product was analyzed via XRD, as depicted in Figure S12b,c. The diffraction peaks observed in the TGA final product from Mo_2_N matched perfectly with α‐MoO_3_ (PDF #01‐074‐7912). On the other hand, The XRD analysis of the final product from VN revealed diffraction peaks corresponding to the V_2_O_5_ phase (PDF #00‐041‐1426). Based on stoichiometry, the TMN content was calculated according to the chemical equation inserted in Figure 2b. It was determined that 65.7 wt% of Mo_2_N in Mo_2_N@hC, 39.4 wt% of VN in VN@hC, 67.3 wt% of Mo_2_N in Mo_2_N@sC, and 48.1 wt% of VN in VN@sC.

In order to monitor the structural changes and the porosity of VN@sC and Mo_2_N@sC treated at different temperatures, N_2_ physisorption analysis was acquired. The adsorption‐desorption isotherms and its pore size distribution of VN@sC and Mo_2_N@sC obtained at different temperatures are shown in Figure 2c–f. IV‐type hysteresis loop can be observed for all samples, suggesting mesoporous structures [48]. However, it reflected a considerable change on the specific surface area determined by Brunauer‐Emmett‐Teller (BET) model as the temperature increases. For instance, the specific surface area of VN@sC increased from 41.8 to 65 m^2^ g^−1^, while the pore volume grew from 0.119 to 0.245 cm^3^ g^−1^ as the temperature increased from 600°C to 1000°C. Meanwhile, for Mo_2_N@sC, the specific surface area increased from 21.7 to 103.5 m^2^ g^−1^, and the pore volume rose from 0.116 to 0.155 cm^3^ g^−1^, respectively. The pore size distribution shows a reorganization of the pore structure, attributed to the formation of additional voids in the carbon matrix as metal domains grow and cluster. The specific surface areas and pore volumes of VN@sC and Mo_2_N@sC are listed in Table S2. TEM observations align well with the observed increase in surface area, highlighting the crystallite rearrangement and growth during thermal treatment. As temperature increases, crystallites gradually coalesce into larger clusters, resulting in a notable restructuring of the porous architecture.

Figure S13a,b shows the N_2_ adsorption‐desorption isotherms and its pore size distribution of Mo_2_N@hC and VN@hC. Based on BET model, the specific surface area was determined to be 142.7 m^2^ g^−1^ and 218.3 m^2^ g^−1^, respectively. Conversely, pore size distribution revealed a dominant small mesoporous domain in both samples, approximately 6  and 4 nm, respectively. This phenomenon may be attributed to the inter‐particle packing of TMN nanoparticles within the carbon matrix. Additionally, both Mo_2_N@hC and VN@hC exhibited a similar distribution in the 20–30 nm range, likely arising from the presence of hollow voids. [36] Although both samples originated from the same hollow PILs template, the notorious difference in specific surface area can be attributed to the inter‐sphere packing pores, influenced by the TMN content and distribution. Raman spectroscopy was applied to quantify the degree of disorder of the carbon sphere. Figure S14 shows the Raman spectra for VN@hC and Mo_2_N@hC. Both samples display a prominent G band, associated with sp^2^‐hybridized carbon, and a D band, indicative of the presence of amorphous carbon. The calculated I_D/G_ ratio for VN@hC was 1.01, suggesting a higher degree of disorder, while the ratio for Mo_2_N@hC was 0.61, indicating fewer defects [49].

A preliminary experiment was carried out to examine the nature of the carbon composition formed during the decomposition of PDA@PILs in the absence of ionic exchange. In this experiment, the sample was heated in an Ar atmosphere to 600°C for 3 h at a heating rate of 3°C min^−1^. To analyze the chemical composition of the resulting bulk carbon, high‐resolution X‐ray photoelectron spectroscopy (XPS) was employed. Figure S15a,b illustrates the XPS scans on C 1s and N 1s, respectively. The C 1s spectrum of the PDA@PILs‐derived carbon is dominated by a main peak at about 284.4 eV, characteristic of sp^2^ hybridized carbon, together with a shoulder at higher binding energy that can be assigned to C–N species [50, 51, 52]. The N 1s envelope is broad and centered in the 398–402 eV region, indicating the coexistence of several N species like pyridinic, pyrrolic, and graphitic nitrogen environments, in agreement with previously reported N doped carbons. [52, 53]

To facilitate the identification of the oxidation states and surface chemical composition containing in each phase, high‐resolution XPS provided a consistent analysis of the TMNs embedded in hollow and solid carbon, as well as the products obtained at different thermal treatments. Figure S16 introduces the VN@hC and Mo_2_N@hC XPS spectra on V 2p, N 1s, C 1s, and Mo 3d, Mo 3d/N 1s, and C 1s, respectively. For VN@hC, the V 2p region (Figure S16a) shows a typical V 2p_3/2_ / V 2p_1/2_ doublet with the main intensity located near 514–515 eV, consistent with V–N bonding in VN, and a weaker high binding energy tail that we tentatively ascribe to a thin surface layer of oxidized vanadium species [54, 55, 56]. The N 1s spectrum (Figure S16b) contains a component at about 397 eV from nitride nitrogen together with broader features at higher binding energies assigned to nitrogen in the carbon matrix (pyridinic, pyrrolic and graphitic N), while the C 1s spectrum (Figure S16c) is dominated by sp^2^ carbon with minor contributions from C–N and C–O species [50, 51, 52, 57]. For Mo_2_N@hC, the Mo 3d spectrum (Figure S16d) displays a Mo 3d_5/2_ / 3d_3/2_ doublet centered around 229–232 eV, characteristic of Mo in a nitride environment, accompanied by a small high binding energy shoulder that presumably indicates the presence of surface Mo–O species. [54, 58, 59] In the Mo 3p/N 1s region (Figure S16e), a clear N 1s signal is observed near 397–401 eV, confirming that nitrogen is present in both, Mo_2_N and a doped carbon framework. The corresponding C 1s spectrum (Figure S16f) again shows mainly sp^2^ carbon with weak higher energy features tentatively ascribed to C–N/C–O bonds, similar to VN@hC [50, 51, 52, 57]. Oxygen species observed in V 2p, Mo 3d, N 1s, C 1s could be attributed to a passivated surface when contacting the oxygen in the atmosphere post‐synthesis rather than to bulk V–O or Mo–O phases. Given the absence of oxygen‐related crystalline signals in the XRD data, it can be assumed that the oxygen content is relatively limited and primarily confined to the surface. Such a passivated layer is common for TMNs and is not thick enough to dominate the electrochemical response. Instead, it provides additional polar sites that can participate in LiPS binding while the underlying nitride controls the electronic structure and charge transfer.

**FIGURE 3:**
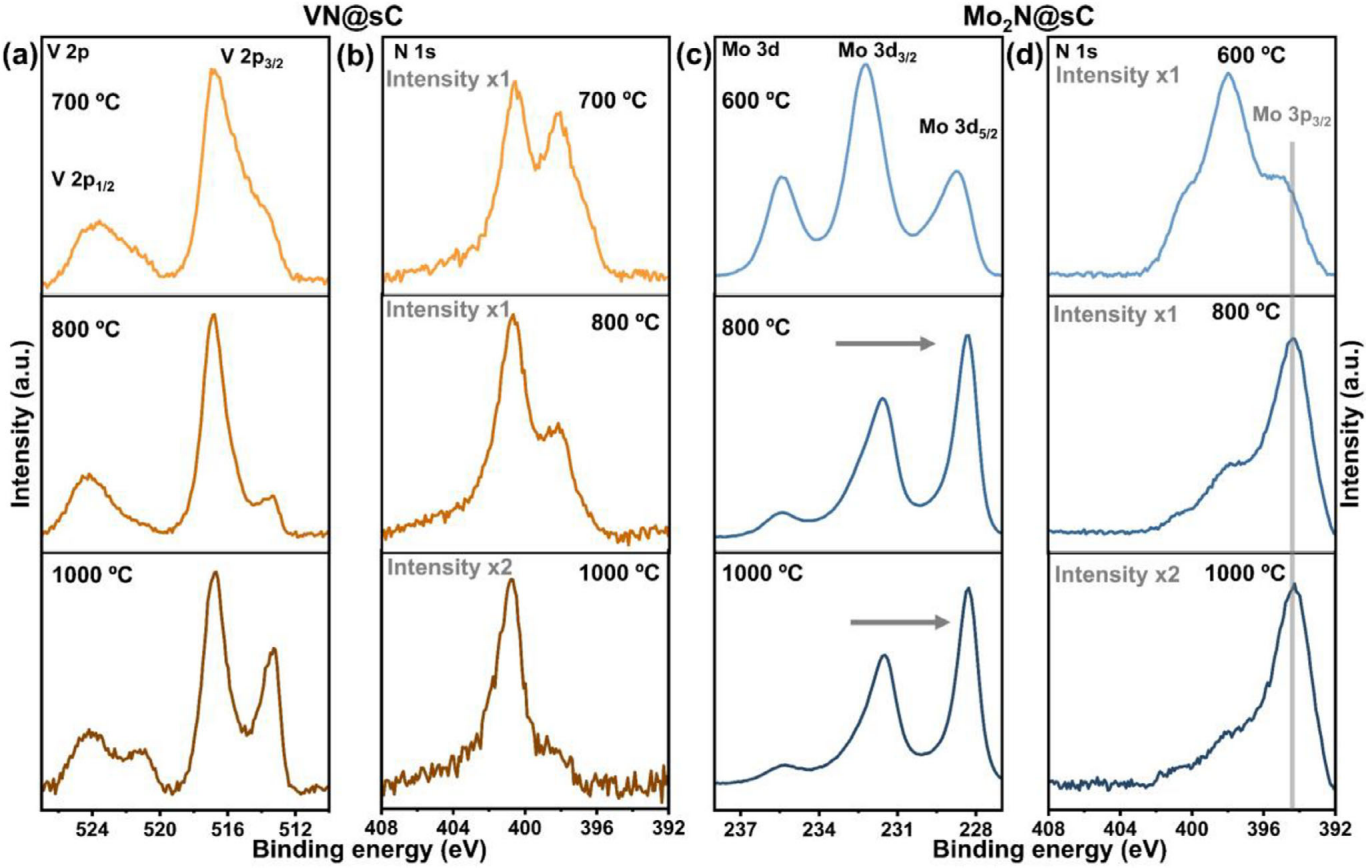
High resolution XPS spectra of V 2p (a) and N 1s (b) of VN@sC acquired at 700°C, 800°C, and 1000°C. High resolution XPS spectra of Mo 3d (c) and N 1s (d) of Mo_2_N@sC acquired at 600°C, 800°C, and 1000°C.

The temperature dependent XPS of VN@sC is shown in Figure 3a,b and Figure S17. The V 2p envelopes (Figure 3a) mainly retain the characteristic VN doublet, but gradually shift to slightly lower binding energies with increasing calcination temperature, indicating progressive reduction of the vanadium centers and partial transformation toward more carbide like environments at high temperature, most prominently indicated by the appearance of the rather narrow V 2p_3/2_ and V 2p_1/2_ features at 513 and 521 eV, respectively. This trend is consistent with the appearance of a low binding energy shoulder in the C 1s spectra (Figure S17a–c) that can be attributed to V–C bonding [60], together with an increased relative intensity of the sp^2^ carbon component, which reflects graphitization of the carbon matrix. Meanwhile, the overall N 1s intensity (Figure 3b) decreases as the temperature increases, showing that nitrogen is gradually expelled from the structure through nitride decomposition and loss of nitrogen functionalities in the carbon.

Similar behavior is observed for Mo_2_N@sC (Figure 3c,d and Figure S18). Except for the 600°C data (which spectral shape can be tentatively explained by a pronounced presence of [surface‐]oxidized Mo), the Mo 3d spectra (Figure 3c) remain characteristic of Mo_2_N but display a noticeable shift toward lower binding energy at higher temperature, which is consistent with the formation of Mo–C species and the onset of carbide formation [58, 61]. In parallel, the N 1s signal in the Mo 3p/N 1s region (Figure 3d) weakens with temperature, indicating nitrogen loss from the nitride phase. The C 1s spectra (Figure S18a–c) show an increasing contribution from graphitic sp^2^ carbon and the emergence of a low binding energy shoulder near 283 eV that can be attributed to Mo–C bonding. Overall, these changes point to progressive reduction of the metal centers, reorganization and depletion of nitrogen, and a transition of the carbon framework toward a more graphitic and partially carbide forming structure upon high temperature treatment.

Although both the specific surface area and distribution of Mo_2_N within the carbon matrix play crucial roles, it is worth noting that homogeneously distributed Mo_2_N with smaller crystallite sizes could potentially enhance the material's performance further. This configuration offers increased surface accessibility for active sites, facilitating better adsorption and reaction kinetics, potentially leading to improved electrochemical performance in Li–S batteries [62]. TMN@C obtained at the minimal temperature required to form the nitride phase were screened for further catalytic studies toward LiPSs due to their homogeneity throughout the carbon matrix of the spheres. Additionally, a bare hollow carbon (hC) material was obtained by removing the TMNs through acid etching under harsh conditions from TMNs@hC and subsequently used as a control in electrochemical evaluations. Figure S19 presents the TEM image of hC after etching the embedded TMNs, along with the XRD patterns characteristic of amorphous carbon and the N_2_ adsorption‐desorption isotherms. The BET specific surface area of the hC was found to be 365 m^2^ g^−1^.

**FIGURE 4:**
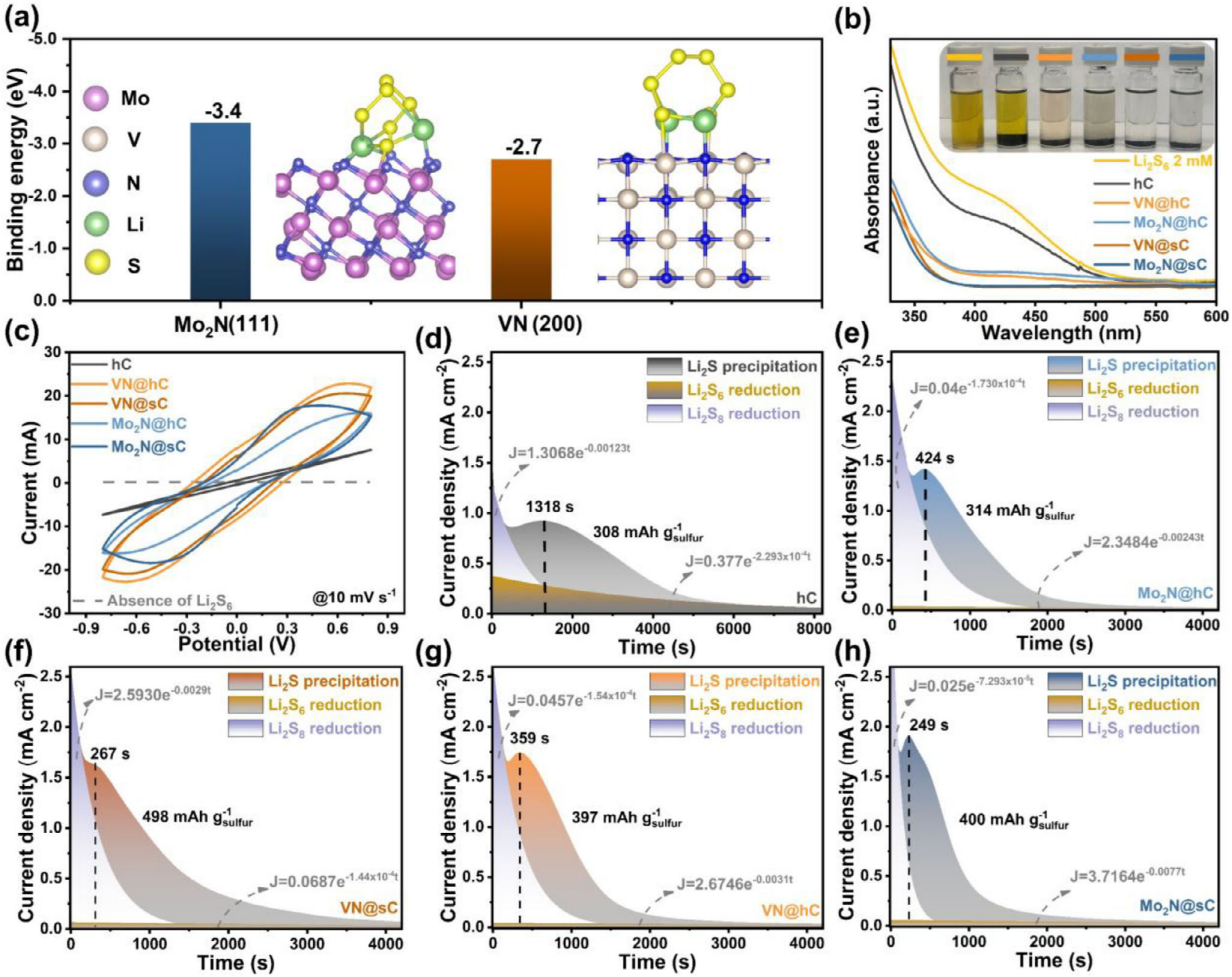
DFT calculations of the binding energy of Li_2_S_6_ on Mo_2_N (111) and VN (200) planes (a). Static adsorption test for the 3 mL of 2 mM Li_2_S_6_ solution against 30 mg of TMNs@C and hC. UV–vis absorption spectra of the corresponding supernatants (b). CV curves of symmetrical cell at 10 mV s^−1^ of the studied materials including the absence Li_2_S_6_ (c). Potentiostatic discharge curves at 2.05 V (vs. Li/Li^+^) for Li_2_S precipitation, hC (d), VN@hC (e), VN@sC (f), Mo_2_N@hC (g), and Mo_2_N@sC (h).

First‐principles simulations based on density functional theory (DFT) were conducted to investigate the binding energies of the most prominent spatial configurations for both TMNs: the Mo_2_N (111) plane and the VN (200) plane, in relation to Li_2_S_6_, which represents a critical intermediate in the dissolution and conversion of sulfur during the discharge process. The calculation details are described in the experimental section. Figure 4a compares the calculated adsorption energies of Li_2_S_6_ on Mo_2_N (111) and VN (200), giving values of −3.4 and −2.7 eV, respectively, which indicates stronger thermodynamic anchoring of Li_2_S_6_ on Mo_2_N. The inset side‐view models of Li_2_S_6_ adsorbed on Mo_2_N (111) and VN (200) surfaces illustrate distinct atomic interactions, as reflected by the different molecular deformations of Li_2_S_6_ and metal‐sulfur bond lengths at the interfaces, which are directly related to the catalytic processability of LiPSs during discharge and charge. The corresponding top‐view configurations are shown in Figure S20. Projected density of states (PDOS) analysis (Figure S21) further reveals that VN exhibits a much higher density of states at the Fermi level, dominated by V 3d states with pronounced overlap with S 3p states, while Mo_2_N shows a lower Mo 4d contribution near the Fermi level and weaker hybridization with S 3p. This combination of moderate Li_2_S_6_ binding on VN together with a more favorable electronic structure facilitates rapid charge transfer and efficient activation of polysulfides, whereas Mo_2_N has deeper binding and less conductive surface, and is more prone to product trapping and passivation. This electronic‐thermodynamic balance would favor the LiPSs conversion and positively influence the electrochemical performance.

DFT was further employed to investigate the sulfur reduction pathways on the surface of the TMNs species. Figure S22 introduces the Gibbs free‐energy landscape for the sulfur reduction reaction from S_8_ to Li_2_S, calculated based on the stepwise formation of soluble polysulfide intermediates and solid Li_2_S_2_/Li_2_S species. Both Mo_2_N (111) and VN (200) exhibit a thermodynamically and kinetically favorable sulfur reduction pathway. Mo_2_N exhibits a more negative free‐energy change, indicative of stronger sulfur adsorption and activation. Although the Li_2_S_8_ → Li_2_S_6_ conversion is thermodynamically downhill on both catalysts, VN maintains a relatively flat free‐energy profile, enabling smoother long‐chain polysulfide conversion, whereas Mo_2_N shows pronounced energy fluctuations along the reaction pathway that may kinetically trap intermediate species. Moreover, the deeper free‐energy minimum for the Li_2_S_2_/Li_2_S step on Mo_2_N suggests a faster Li_2_S nucleation compared with VN.

Furthermore, to observe the static anchoring toward Li_2_S_6_, the adsorption test was performed by monitoring the supernatant obtained by adding 30 mg of sample into 3 mL of 2 mM Li_2_S_6_ (in DME/DOL 1:1 v/v) solution, kept undisturbed for 7 h. Figure 4b presents the UV–vis absorption spectra of the supernatants. Li_2_S_6_ solution shows a yellowish tone as observed in the inset, exhibiting two main absorption bands in the spectrum at 340 and 410 nm, normally referred to S_3_^2−^ and S_4_^2−^, respectively [63]. The supernatant of the samples containing TMNs nanoparticles changed from yellow to nearly colorless, indicating strong adsorption capability in extracting LiPSs from solution to the solid matrix. In contrast, the supernatant with metal‐free hC remained lightly yellow, suggesting a poor affinity for LiPSs. Despite variations in TMN type, the solid carbon architecture resulted in a clearer supernatant, likely due to increased accessibility to polar sites. This is well supported by the UV–vis absorption spectra, which show a notable fading of the main absorption band.

Taken together, the adsorption tests and DFT results clarify why VN outperforms Mo_2_N in specific configurations. The UV–vis spectra show that introducing TMNs into the carbon framework dramatically enhances Li_2_S_6_ uptake compared with metal free hC, confirming that both Mo_2_N and VN provide abundant polar sites for static anchoring of LiPSs. Mo_2_N induces a slightly stronger decoloration, consistent with its more negative Li_2_S_6_ adsorption energy of −3.4 eV relative to −2.7 eV on VN (200). However, PDOS analysis reveals that VN possesses a higher density of states at the Fermi level and stronger V 3d‐S 3p hybridization, which facilitates charge transfer and activation of adsorbed Li_2_S_6_. In combination with the smoother Gibbs free energy landscape on VN, this leads to faster conversion of anchored LiPSs and mitigates surface passivation, whereas Mo_2_N tends to over stabilize intermediates on a less conductive surface.

The catalytic effect of the host materials on the conversion of LiPSs was evaluated using cyclic voltammetry (CV) in a symmetrical cell at 10 mV s^−1^, as illustrated in Figure 4c. In the absence of Li_2_S_6_, the symmetrical cell displayed only minimal capacitive current, highlighting the lack of significant redox activity. However, when Li_2_S_6_ was introduced, the polarization profiles observed for bare hC and TMNs containing electrodes reflected indeed a redox activity of Li_2_S_6_. Notably, the current signal increased significantly with TMNs containing electrodes indicating enhanced electrochemical conversion of LiPSs. Interestingly, despite the differing architectures, the samples containing VN nanoparticles exhibited the highest catalytic effect, as reflected by their larger CV profile areas, which may indicate greater charge transfer and enhanced electrochemical activity. To exclude the influence of different TMN loading amounts on these electrochemical responses, additional symmetrical‐cell CV measurements were performed at a lower scan rate of 1 mV s^−1^ (Figure S23) with the current normalized to the mass of active TMNs. VN@hC displays reduction/oxidation peaks at −0.204 V/0.198 V, while VN@sC exhibits peaks at −0.152 V/0.173 V with higher currents. For Mo_2_N electrodes, Mo_2_N@hC shows peaks at −0.210 V/0.289 V, and Mo_2_N@sC at −0.312 V/0.291 V with higher currents. The results demonstrate that VN is more electrocatalytically active than Mo_2_N, and that solid carbon supports slightly enhance the accessibility of TMN active sites, resulting in higher redox currents. Moreover, under these mass‐normalized conditions, clear differences in electrochemical behavior were still observed, confirming that the enhanced catalytic performance originates predominantly from the intrinsic catalytic activity of the TMNs and their synergistic interaction with the architected carbon supports.

Figure 4d–h presents the investigation of Li_2_S nucleation conducted using the potentiostatic discharge method. The reduction of LiPSs was first carried out uniformly via galvanostatic discharge at 0.1 C, with a cutoff voltage of 2.16 V (vs. Li/Li^+^). This voltage is sufficiently high to prevent premature Li_2_S nucleation while remaining low enough to facilitate the reduction of higher‐order LiPSs. Subsequently, potentiostatic discharge was performed at 2.05 V (vs. Li/Li^+^) to precisely control the overpotential. This potential is low enough to overcome the energy barrier for Li_2_S nucleation, enabling the formation and continuous growth of solid Li_2_S over time [64]. The pronounced enhancement of the discharge peak, corresponding to Li_2_S nucleation in TMN‐containing samples, directly reflects the improved electrochemical conversion of LiPSs into solid Li_2_S, implying faster nucleation and growth kinetics. As a result, the nucleation peak for Li_2_S appeared in a shorter timeframe: 249 and 424 s for Mo_2_N@sC and Mo_2_N@hC, respectively, and 267 and 359 s for VN@sC and VN@hC, respectively. These values are significantly shorter than the 1318 s observed for hC alone. Consequently, this enhancement in kinetics significantly improves overall energy storage, as quantified by Faraday's law. The area under the curve represents the capacity associated with the nucleation and growth of Li_2_S, obtained by fitting and subtracting two exponential decay functions that are associated to the reduction of LiPSs, as indicated in Figure 4d–h. The determined discharge capacities are 397 mAh g^−1^ for VN@hC, 498 mAh g^−1^ for VN@sC, 314 mAh g^−1^ for Mo_2_N@hC, and 400 mAh g^−1^ for Mo_2_N@sC, all of which demonstrate superior performance compared to the bare hollow carbon (hC) electrode, which has a discharge capacity of 308 mAh g^−1^. Meanwhile, these findings also indicate that the solid dense carbon spheres embedded with TMNs exhibit enhanced Li_2_S nucleation. The observed differences can be correlated to both the higher TMN content and denser packing, which collectively enhance the surface accessibility. This increased accessibility could play a crucial role in facilitating faster and more efficient nucleation processes in nanostructured TMNs.

In order to evaluate the electrochemical performance, the as‐synthesized TMN@hC and TMN@sC were used as a host material for sulfur. All the materials were prepared by conventional sulfur infusion methods, as detailed in the experimental part. TGA in argon atmosphere was employed to confirm the successful impregnation of sulfur into the host materials. Figure S24 shows the weight‐loss curves of all the materials used for cell assembly. A residual mass of approximately 30 wt% of the initial weight was observed, indicating 70 wt% of sulfur. The as‐formed host material‐sulfur composite was used as active material in Li–S coin cells.

**FIGURE 5:**
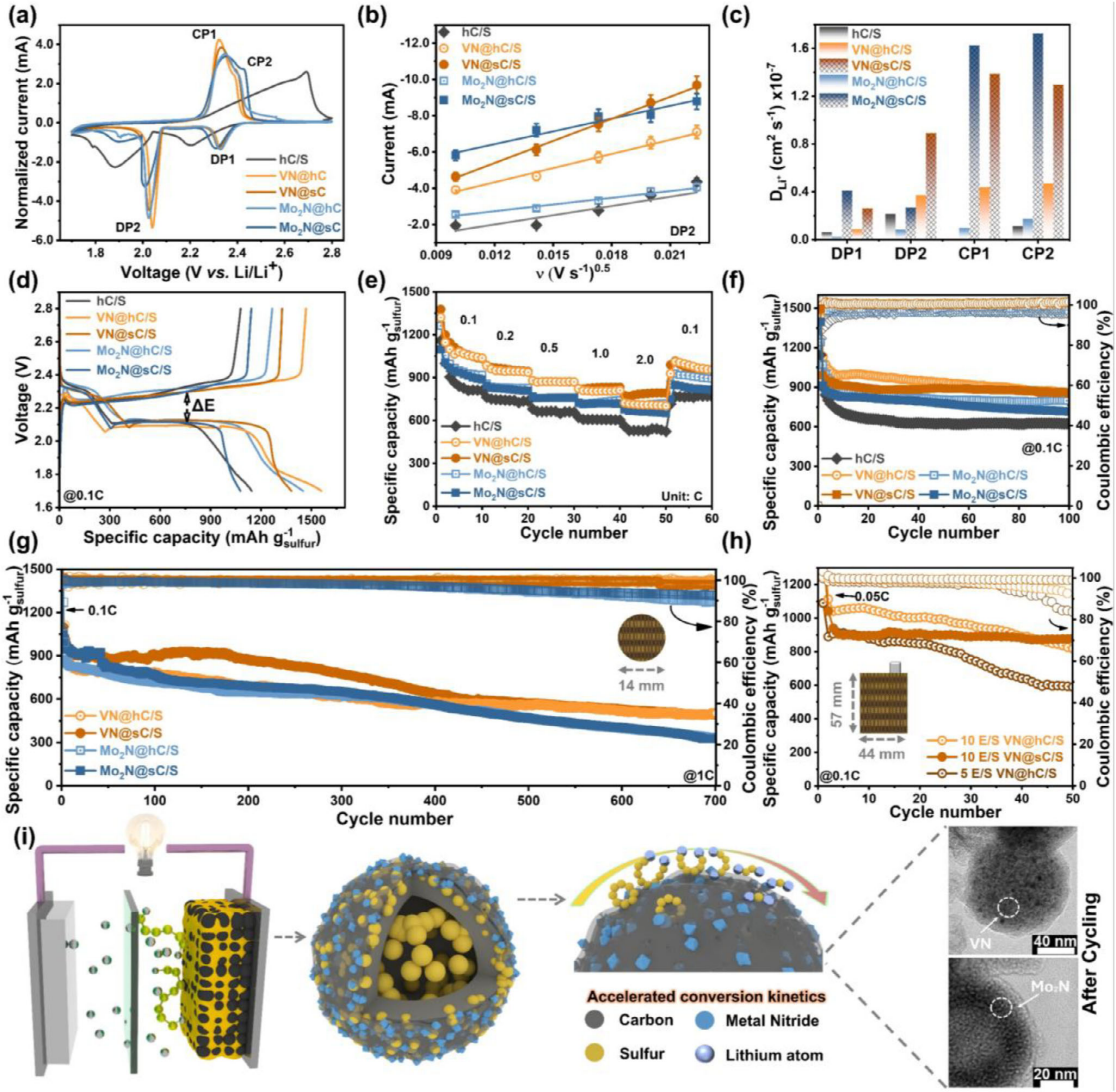
Electrochemical performance of TMN‐embedded in hollow and solid carbon hosts for Li–S cells including bare hC (sulfur content of 49 wt%). Cyclic voltammetry (CV) curves scanned at 0.1 mV s^−1^ (a), DP2 fitting curves for estimating $D_{Li^{+}}$ (b), summary of $D_{Li^{+}}$ for all the electrochemical events (c), 0.1 C discharge‐charge profiles (1 C = 1675 mA g^−1^) (d), rate capability from 0.1 to 2 C (e), cycling stability at 0.1 C (f), long‐term cycling stability at 1 C (Capacity decay per cycle: Mo_2_N@sC/S: 0.1%, Mo_2_N@hC/S: 0.09%, VN@sC/S: 0.08%, VN@hC/S: 0.07%) (g), cycling stability of VN@C/S (sulfur loading of 2 mg cm^−^^2^) pouch cells with an E/S ratio of 10 µL mg^−1^ and VN@hC/S with an E/S ratio of 5 µL mg^−1^ at 0.1 C (Capacity retention after 50 cycles: 10 E/S VN@sC/S: 85%, 10 E/S VN@hC/S: 75%, 5 E/S VN@hC/S: 67%) (h). Illustration of the mechanism of nanostructured TMNs on optimized electrochemistry in Li–S pouch cells, underscoring the enhanced kinetics, catalytic effect and robustness (i).

Figure 5a introduces the CV curves recorded in a voltage range of 1.7–2.8 V (vs Li/Li^+^) at a scanning rate of 0.1 mV s^−1^. The extensive analysis of the catalytic activity is shown in Figure S25. The hC/S displays typical reduction peaks at approximately 2.2 V and 1.89 V during the cathodic scan. These peaks correspond to the conversion of octatomic sulfur to long‐chain LiPSs (Li_2_S_x_, 4 ≤ x ≤ 8) and subsequently to short‐chain products (Li_2_S_2_/Li_2_S), respectively. The anodic scan also unveils a distinct oxidation shoulder, emblematic of the intricate electrochemical transformations attributed to the conversion of (Li_2_S/Li_2_S_2_) to long‐chain LiPSs. The conversion process entails a series of complex intermediate steps, including the dissolution and recombination of LiPSs species. Following the nuanced conversion process, the curve exhibits an oxidation peak at 2.69 V, signifying the octatomic sulfur nucleation. The pronounced emergence of octatomic sulfur underscores a localized and rapid oxidation event, emphasized by the peak's magnitude [65, 66]. On the other hand, the introduction of TMNs provided a catalytic effect observed in both cathodic and anodic scans. During the cathodic scan, the presence of TMNs results in a noticeable positive shift in both reduction peaks. This shift may indicate a reduction in overpotential, suggesting that the conversion of sulfur to lithium polysulfides (LiPSs) and subsequently to Li_2_S is occurring more readily. Additionally, the enhanced kinetics facilitated by TMNs lead to a more localized and uniform conversion of the reductive species, as reflected by the higher intensity of the reduction peaks [67]. Similarly, in the anodic scan, TMNs contribute to more pronounced and well‐defined oxidation peaks. Additionally, a negative shift in these peaks was observed, suggesting enhanced overpotential as well. The influence of TMN content played an important role in the CV experiment. For all the samples containing TMNs, the CV curves exhibited noticeable shifts along with a well‐defined oxidation peak around 2.3 V. This peak suggests a more localized and rapid conversion of lower‐order LiPSs into long‐chain LiPSs.

To further evaluate the Li^+^ diffusion coefficient (D_L__i_^+^) for the distinct redox peaks, the Randles‐Sevcik equation, as described in Equation I in the Supporting Information, was applied to analyze the CV curves collected at various scan rates, ranging from 0.1 to 0.5 mV s^−1^ [7], as illustrated in Figure S26. The relationship between the peak current at various electrochemical events and the square root of the scan rate is depicted in Figure S27. This correlation provides insights into the kinetics of Li^+^ ion diffusion within the electrode material. As observed in the CV curves (Figure 5a), four main events are observed in the overall electrochemical process. DP1 represents the conversion of octatomic sulfur to long‐chain LiPSs. DP2 corresponds to the reduction of LiPSs to Li_2_S, a critical step that contributes significantly to the overall capacity in Li‐S systems, therefore, included in Figure 5b. It turned out that VN nanoparticles embedded in carbon architecture exhibit the highest D_L__i_^+^ for DP2, with values of 0.90 × 10^−7^ cm^2^ s^−1^ for VN@sC and 0.37 × 10^−7^ cm^2^ s^−1^ for VN@hC. CP1 is associated with the localized conversion of short‐chain LiPSs into other long‐chain species. Finally, CP2 corresponds to the transformation of long‐chain LiPSs back to octatomic sulfur. The calculated D_L__i_^+^ are summarized in Figure 5c. It can be observed that the diffusion‐controlled nature of TMNs is highly dependent on both the matrix morphology and composition. The highest overall $D_{Li^{+}}$ during charge events was observed for VN@sC and Mo_2_N@sC. For CP1, the $D_{Li^{+}}$ values were 1.40 × 10^−7^ and 1.62 × 10^−7^ cm^2^ s^−1^, respectively, while for CP2, the $D_{Li^{+}}$ values were 1.31 × 10^−7^ and 1.71 × 10^−7^ cm^2^ s^−1^, respectively. The enhanced diffusion behavior can be attributed to the confined structural characteristics of these materials, including the interaction between the metal nitrides and the carbon matrix, which facilitates efficient ion movement.

Tafel analysis of CV curves at 0.1 mV s^−1^ (Figure S28a,b) reveals slopes of about 60–80 mV dec^−1^ for the S_8_ → Li_2_S_x_ step and 20–30 mV dec^−1^ for Li_2_S_x_ → Li_2_S, confirming different kinetics for the two reduction stages. Using hC/S as a reference, we extracted relative activation energy differences (ΔE_a_) from the Tafel plots (Figure S28c). The resulting profiles (Figure S28d) show that the Li_2_S_x_ to Li_2_S conversion has a higher kinetic barrier than the initial sulfur reduction and is therefore rate limiting. For the S_8_ → Li_2_S_x_ step, VN@hC shows the largest ΔE_a1_ (59.3 kJ mol^−1^), followed by VN@sC (55.3 kJ mol^−1^) and Mo_2_N@sC (56.8 kJ mol^−1^), whereas Mo_2_N@hC (36.1 kJ mol^−1^) gives the smallest value. This indicates that VN benefits most from the hollow carbon scaffold and is the most efficient for long chain polysulfide conversion, while Mo_2_N in the hollow structure provides only moderate acceleration at this stage. For the Li_2_S_x_ → Li_2_S step, the highest ΔE_a2_ values are obtained for Mo_2_N@hC (213.6 kJ mol^−1^) and VN@hC (201.5 kJ mol^−1^), significantly above VN@sC (178.6 kJ mol^−1^) and Mo_2_N@sC (148.8 kJ mol^−1^). This shows that the hollow carbon architecture greatly lowers the barrier for Li_2_S nucleation for both TMNs, with Mo_2_N gaining a slight advantage in the short chain polysulfide to Li_2_S conversion. Overall, these trends demonstrate that VN coupled with hollow carbon is particularly effective for long chain LiPS conversion, whereas Mo_2_N in the hollow scaffold preferentially promotes the late Li_2_S_x_ → Li_2_S step, which is consistent with the DFT free energy landscapes and reveals a clear structure‐kinetics relationship between TMN type, carbon morphology and polysulfide conversion.

To evaluate the electrochemical response of VN@hC and VN@sC, the CV curves were collected at higher scanning rate of 3 mV s^−1^, as illustrated in Figure S29. Compared to low scanning rate, where the Li^+^ gives more time to diffuse to and from the electrode surface, high scanning rate gives less time to diffuse, and the system is limited by the rate of charge transfer at the electrode surface. The fast response of both samples is reflected in the electrochemical events observed during the anodic and cathodic scans. VN@sC exhibited a higher current peak, suggesting a faster response, which indicates that VN@sC has more efficient and controlled kinetics, leading to improved electrochemical performance at higher current densities. Figure 5d introduces the first galvanostatic charge‐discharge profile of all the host materials recorded at 0.1 C. A clear capacity density change is observed with TMNs‐based host materials, presenting higher sulfur utilization compared to that of hC. Moreover, a hysteresis voltage of around 0.18 V was determined at half of the cell capacity. This relatively low hysteresis voltage suggests reduced energy losses and polarization during the charge‐discharge process. Figure S30 shows the Nyquist plots of all the materials obtained by Electrochemical impedance spectroscopy (EIS) performed at open‐circuit‐potential. The charge‐transfer region occurring at high frequencies is depicted in the inset, displaying a rounded‐single semicircle in all the samples. Specifically, the sulfur cathode prepared with hC exhibits a charge‐transfer resistance of 50 ohms at the electrode interfaces. In contrast, cathodes prepared with TMNs host materials disclosed lower charge transfer resistance values of approximately 40 ohms for Mo_2_N@sC, 37 ohms for VN@sC, 36 ohms for Mo_2_N@hC, and 30 ohms for VN@hC. These findings indicate a notable improvement in charge transfer kinetics, attributed to the enhanced catalytic activity and superior electrical conductivity of TMNs.

*Operando* Raman was further used to track sulfur speciation during the first discharge of VN@hC/S and a Ketjenblack control (KB/S). For KB/S (Figure S31a,b), the initial S_8_ bands at 152, 218, and 470 cm^−1^ fade rapidly, while the long‐chain Li_2_S_x_ (x = 6–8) feature at 415 cm^−1^ appears and then persists throughout discharge, indicating that soluble polysulfides remain and are not efficiently converted. In contrast, VN@hC/S (Figure S31c,d) shows a brief 415 cm^−1^ signal but rapidly develops a dominant band at 453 cm^−1^ with a minor peak near 275 cm^−1^, assigned to intermediate‐chain Li_2_S_x_ (x = 3–5), evidencing accelerated long‐to‐intermediate conversion. The 453 cm^−1^ band intensifies during discharge but decreases markedly below 1.9 V, and polysulfide‐related signals nearly vanish at the end of discharge, consistent with further conversion to solid Li_2_S. The heatmaps (Figure S31b,d) highlight this contrast, confirming more complete polysulfide conversion enabled by VN@hC, in agreement with the DFT adsorption results.

Figure 5e presents the rate capability acquired at 0.1, 0.2, 0.5, 1, and 2 C. It was found that the cells prepared using TMNs‐based host materials demonstrated superior performance with remarkable reversibility after turning back to 0.1 C across all tested rates compared to the cell with hC‐based cathodes. The sulfur retention at 2 C for samples containing TMNs demonstrated specific capacities of 657 mAh g^−1^ for Mo_2_N@sC/S, 715 mAh g^−1^ for both Mo_2_N@hC/S and VN@hC/S, and 792 mAh g^−1^ for VN@sC/S. In contrast, the hC/S sample delivered only 525 mAh g^−1^. The enhanced stability at higher current densities can be attributed to the strong confinement of LiPSs and the catalytic activity of TMNs, which collectively promote efficient and rapid redox reactions during the discharge‐charge process. Additionally, it was observed that there is a clear difference in stability between VN and Mo_2_N, indicating that the catalytic effect of each metal plays a significant role in converting and adsorbing LiPSs species. Figure 5f shows the cycling stability at 0.1 C over 100 cycles, with initial specific capacities of 1390 mAh g^−1^ for Mo_2_N@sC/S, 1250 mAh g^−1^ for Mo_2_N@hC/S, 1425 mAh g^−1^ for VN@hC/S, 1490 mAh g^−1^ for VN@sC/S, and 1140 mAh g^−1^ for hC/S. After 100 cycles, the specific capacity retention was 713 mAh g^−1^ for Mo_2_N@sC/S, 790 mAh g^−1^ for Mo_2_N@hC/S, 855 mAh g^−1^ for both VN@hC/S and VN@sC/S, and 630 mAh g^−1^ for hC/S. This demonstrates the robust performance of the modified samples, in contrast to bare hC, which showed more pronounced capacity fading after only a few cycles. To assess the structural durability of the host materials, cycling stability studies were done at 0.5 C for 200 cycles, as shown in Figure S32. The specific capacities delivered after 200 cycles at 0.5 C disclosed values of 625 mAh g^−1^ for Mo_2_N@sC/S, 650 mAh g^−1^ for Mo_2_N@hC/S, 709 mAh g^−1^ for both VN@hC/S and VN@sC/S, and 540 mAh g^−1^ for hC/S. After cycling, coin cells containing VN@sC and Mo_2_N@hC were selected for disassembly. The Figure S33 exhibits the TEM images of VN@sC and Mo_2_N@hC after cycling, respectively. As observed, both TMNs embedded in the carbon spheres remain clearly visible without any apparent damage, indicating a high degree of structural integrity and stability of the materials after extended cycling.

Figure 5g presents the long‐term cycling at 1 C for 700 cycles to assess the cycling stability at higher current density of TMNs embedded in carbon spheres. Coin cells were initially activated at 0.1 C for one cycle and then continued at 1 C for the following cycles. The specific capacities delivered after 700 cycles at 1 C disclosed values of around 330 mAh g^−1^ for both Mo_2_N@sC/S and Mo_2_N@hC/S, and around 500 mAh g^−1^ for both VN@hC/S and VN@sC/S. The results demonstrated that coin cells sustained robust capacity levels and exhibited stable cycling behavior over 400 cycles. However, the Mo_2_N‐containing electrodes exhibited a sporadic capacity decay in the subsequent cycles, possibly due to material degradation or unwanted interactions with battery components, such as the electrolyte, due to the high binding energy. Interestingly, VN@sC demonstrated superior sulfur utilization and stability compared to the other samples. To further investigate its performance, the VN@sC/S sample was subjected to a higher current density of 2 C, as depicted in Figure S34. Remarkably, the capacity retention of 500 mAh g^−1^ remained elevated to nearly 650 cycles, highlighting its excellent long‐term cycling stability under high‐rate conditions. Table S4 summarizes the electrochemical performance of various VN‐ and Mo_2_N‐based materials reported in the literature, alongside the results obtained in this work. This comparison highlights that our cells deliver competitive cycle stability and capacity retention.

To assess the scalability of the system from coin cells to larger formats, a preliminary single‐layer pouch cell was assembled. For this experiment, a VN@hC/S and VN@sC/S single‐layer pouch cells were assembled using a 4.4 × 5.7 cm^2^ electrode sheet prepared with VN@C as host materials, identical to that used for the coin cells assembly (sulfur loading of 49 wt% in the cathode composition), and an electrolyte‐to‐sulfur ratio (E/S) of ca. 5 and 10 (µL mg^−1^). In Figure 5h the pouch cell cycle stability is presented at 0.1 C. The activation of the cells was done by cycling at 0.05 C once, then continued at 0.1 C. The VN@hC/S pouch cell operated at lean electrolyte conditions (5 µL mg^−1^) showed an initial discharge capacity of 900 mAh g^−1^ and delivered 590 mAh g^−1^ after 50 cycles. On the other hand, the pouch cell with improved electrode wetting (10 µL mg^−1^) exhibited an initial capacity of 1042 mAh g^−1^ for VN@hC/S, which was retained at 819 mAh g^−1^ after 50 cycles. Similarly, VN@sC/S demonstrated an initial capacity of 1040 mAh g^−1^, sustaining 878 mAh g^−1^ after 50 cycles. Extended cycling validation of VN@sC/S further confirmed its stability, maintaining a capacity of 828 mAh g^−1^ after 100 cycles, as shown in Figure S35.

The electrochemical behavior of TMNs@C observed in this Li–S electrochemistry research suggests that nanostructured TMN@sC would exhibit a confined catalytic environment in the conversion reaction of LiPSs, where polysulfides would not entirely diffuse in the interior of the limited porous structure and mostly undergo redox reactions on the surface of active sites. On the other hand, the TMNs@hC hosts offer internal space that accommodates LiPSs conversion reactions within their structure. Despite these structural differences, the open versus confined catalytic environments and the interactions between TMNs and LiPSs have led to varied electrocatalytic and electrochemical performance results [36, 62]. Based on a series of electrochemical tests, it can be inferred that hollow architectures perform reliable at low current densities. This enhanced performance is attributed to their ability to facilitate polysulfide reactions and accommodate volume expansion within their internal space. The solid sphere architecture exhibits higher LiPSs deposition, improved rate performance, and reduced polarization overpotential as evidenced by CV curves. This suggests that solid structures may offer certain features such as shorter ion/electron transport distances, leading to localized kinetics and a confined electrochemical reaction interface. Regarding the class of nanostructured TMNs, although Mo_2_N has a stronger theoretical binding energy, it shows poorer actual electrochemical performance in this experimental setup. Conversely, VN@hC exhibits superior performance at low current densities due to its structure, while VN@sC is preferred for stability and performance at higher current densities. The TMN content in VN@sC contributes to faster reaction kinetics and more efficient use of the electrolyte. Figure 5i highlights the effect of nanostructured TMNs embedded in different carbon architectures for accelerating the electrochemical events in Li–S coin cells, underscoring the enhanced kinetics, catalytic effect and robustness.

**FIGURE 6:**
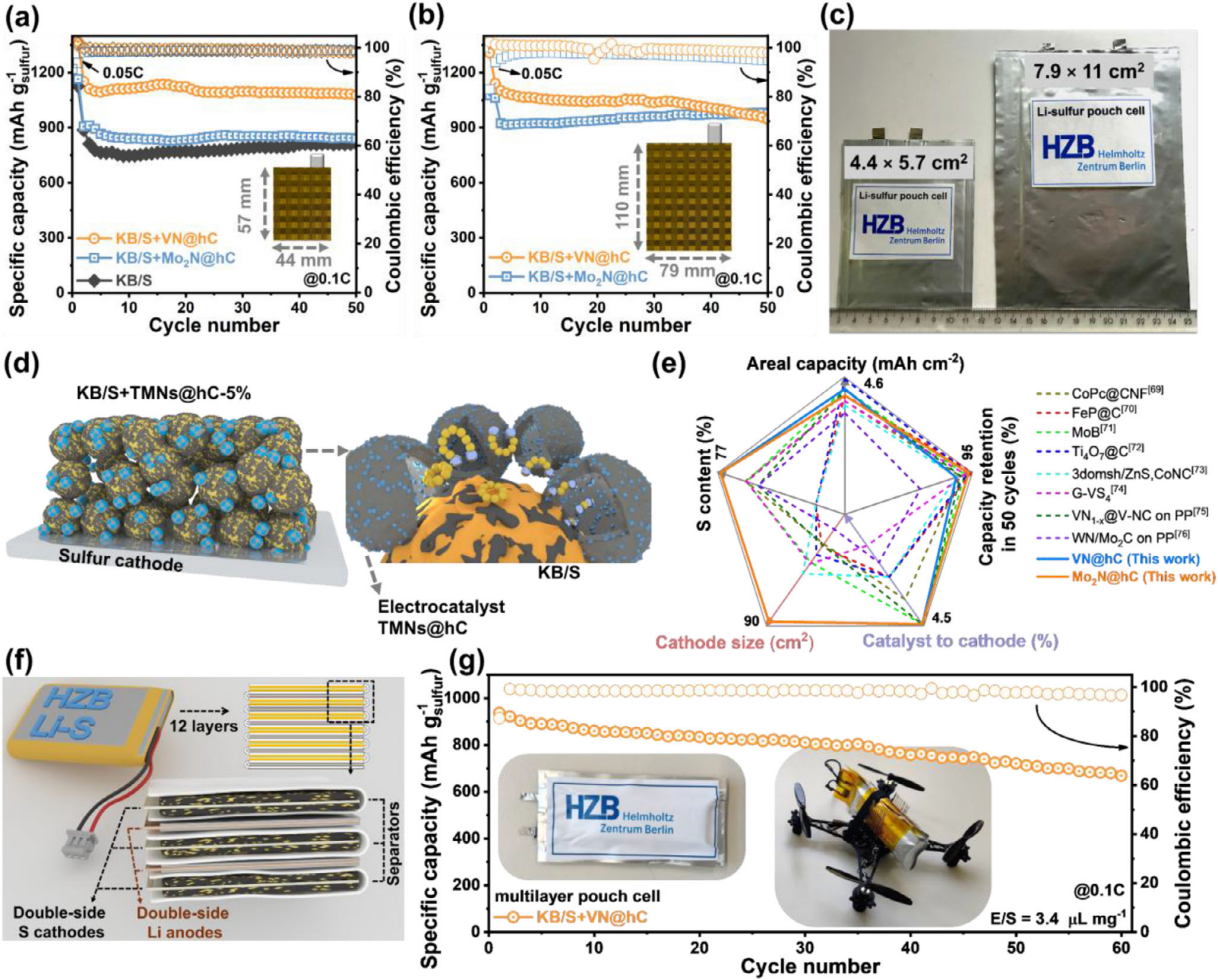
Cycling stability of single‐layer pouch cells (sulfur loading of 3.3 mg cm^−^^2^) with 5 wt% TMNs@hC as an electrocatalyst, an E/S ratio of 10 µL mg^−1^, and a current rate of 0.1 C. 4.4 × 5.7 cm^2^ single‐layer cells, including a reference sample without an electrocatalyst (a). 7.9 × 11 cm^2^ single‐layer cells (b). Photograph of assembled pouch cells (c). Illustration of the role of limited amount of nanostructured TMNs as electrocatalyst in Li–S pouch cells (d). Comparison of electrochemical performances between VN@hC and Mo_2_N@hC electrocatalyst in cathode and other state‐of‐the‐art sulfur host/separator modified with metal compound catalysts in pouch cell scale [69, 70, 71, 72, 73, 74, 75, 76] (e). Pouch cell assembly sketch (f). Electrochemical validation of 12‐layers pouch cell (sulfur loading of 3.6 mg cm^−2^) via cycling stability at 0.1 C and E/S ratio of 3.4 µL mg^−1^. Inset: Picture of the twin pouch cells placed in the custom‐built drone (g).

Considering the effect of the manufacturing concerns such as excellent quality of the cathodes, higher sulfur loading, and lower metal additives amount (TMNs in the battery system), a more realistic approach to sulfur cathode design was further developed, using VN@hC and Mo_2_N@hC as electrocatalyst additives within the sulfur cathodes. This approach enabled a significant reduction of TMN content in the cathode, from around 21% to around 5% by weight, while achieving a higher sulfur areal loading of ca. 3.3 mg cm^−2^ and a higher sulfur content of 72.7 wt% in the cathode coating (w/o Al foil). Figure 6a compares the cyclability of pouch cells with 4.4 × 5.7 cm^2^ single‐layer cathodes operated at 0.1 C under an E/S ratio of 10 µL mg^−1^ with TMNs@hC as electrocatalyst (VN@hC‐5% and Mo_2_N@hC‐5%). Additionally, a control pouch cell was assembled in the same conditions but using pure KB/S cathodes without TMN additives. When Mo_2_N@hC‐5% was used as electrocatalyst the initial specific capacity delivered 910 mAh g^−1^, retaining 850 mAh g^−1^ after 50 cycles. It turns out that the capacity showed a substantial increase when electrocatalyst were existing, even at a relatively low amount of material, indicating that the configuration of TMNs@hC enables a higher sulfur utilization, while the reference sample showed an initial specific capacity of 880 mAh g^−1^and 819 mAh g^−1^ after 50 cycles. In contrast to the VN@hC/S configuration (sulfur host), optimizing the amount and distribution of VN@hC within the system proved to be a critical factor in achieving higher performance with consistent cyclability. On the other hand, the type of electrocatalyst (VN@hC or Mo_2_N@hC) plays a crucial role in optimization, as VN@hC enabled better sulfur utilization even at low metal content within the battery system. Figure 6b introduces the cyclability of a larger size (7.9 × 11 cm^2^) single‐layer pouch cell operated at 0.1 C with an E/S ratio of 10 µL mg^−1^, evaluating the role of TMNs@hC as electrocatalyst in a scaled pouch cell system. Figure 6c shows a photo of the pouch cells with the different sizes used for this testing.

The initial specific capacity of VN@hC‐5% revealed a value of 1143 mAh g^−1^ and a specific capacity of 950 mAh g^−1^ after 50 cycles. This system is comparable to that of its homologous counterpart in a prototype‐sized cell, highlighting the effective scalability and performance retention of the VN@hC architecture. On the other hand, Mo_2_N@hC‐5%, although less effective than VN@hC in preliminary tests, exhibited an initial specific capacity of 1060 mAh g^−1^ and remarkable cyclability, retaining capacity of 980 mAh g^−1^ after 50 cycles.

This scalability underscores the robustness of the TMNs@hC design and its potential for integration into more realistic Li–S battery systems. These configurations show improved overall capacity and demonstrate the potential of nanostructured TMNs to maintain high performance with minimal additive content, while scaling the sulfur cathode. To investigate the structural stability of the cathode materials, post‐mortem analyses were conducted. Figure S37 presents the XRD pattern obtained after cycling, disassembly, and recovery of a KB/S+VN@hC pouch cells (110 mm × 79 mm) after 50 cycles. The active material was gently scraped from the aluminum current collector and subsequently rinsed to remove residual sulfur‐containing species. The diffraction patterns of the cycled VN@hC overlap well with those of pristine VN and show no additional oxide reflections, confirming retention of the crystalline nitride phase without detectable bulk oxidation.

Synchrotron X‐ray absorption spectroscopy (XAS) further corroborates the bulk stability of both Mo_2_N and VN, as XAS probes deeper into the material, making subtle surface changes noticeable in the averaged spectra. Figure S38a introduces the Mo L_3_‐edge XAS spectra of pristine Mo_2_N@hC state and after electrochemical cycling in a coin cell for 100 cycles at 0.1C compared to the spectra measured on commercially available Mo foil and MoO_3_ references. The edge (maximum) positions of the reference compounds are 2522.4 ± 0.1 eV for Mo foil (i.e., elemental Mo, Mo^0^) and 2525.1 ± 0.1 eV for MoO_3_. As the nominal Mo oxidation state of the Mo_2_N@hC samples is assumed to be +2, their edge (maximum) position (i.e., 2524.0 ± 0.1 and 2522.5 ± 0.1 eV for the Mo_2_N powder and the Mo_2_N electrode, respectively) is expected to lie between those of the two reference Mo compounds. An increase in white‐line intensity is seen for the Mo_2_N electrode, an effect that has been ascribed in literature to a rise in unoccupied d‐states and localized changes in metal coordination at the surface [68]. Similar results can be observed when comparing V K‐edge XAS spectra of VN@hC samples, as shown in Figure S38b. The pre‐edge position of the reference compounds are 5464.6 ± 0.1 eV for V foil (i.e., elemental V, V^0^) and 5469.4 ± 0.1 eV for V_2_O_5_ (i.e., V with a +5 oxidation state, V^5+^). As the nominal V oxidation state of the VN@hC samples is +3, their pre‐edge position (i.e., 5469.1 ± 0.1 and 5468.5 ± 0.1 eV for the VN powder and VN electrode, respectively) is again expected to be found between those of the two reference V compounds. Despite a subtle increase in white‐line intensity, no evidence of bulk oxide formation or significant phase transformation was observed. The latter is consistent with the preserved nitride crystal structure identified in the post‐mortem XRD analysis.

Scanning electron microscopy (SEM) was employed to investigate the electrode surface changes in the largest pouch‐cell format. Figure S39a presents the sulfur cathode incorporating VN@hC as the electrocatalyst prior to electrochemical cycling, serving as a reference state. The post‐cycling morphological stability of the cathode is subsequently examined in Figure S39b. Notably, no significant morphological degradation or structural alteration is observed in the active material. Figure S39c,d shows photographs of the electrodes after cycling. In contrast to the cathode, the lithium anode is completely consumed and exhibits a highly uneven surface morphology, suggesting that lithium degradation plays a dominant role in capacity fade modes. On the other hand, the post‐mortem cathode largely preserves its structural integrity, despite minor material detachment, further confirming that cathode degradation is not the primary failure mechanism in this cell configuration.

Figure 6d underscores the pivotal role of the lean TMNs@hC electrocatalyst in enabling practical sulfur cathodes. A trade‐off effect was observed with the hollow structures, which ensured high sulfur utilization even with minimal amounts of TMNs in the pouch cell system. Such hollow carbon architecture promotes the open spatial distribution of active sites, which not only supports more efficient reactions but also enables more straightforward and higher sulfur loading. The strategic design not only facilitates a larger cathode size but also ensures outstanding capacity retention. Figure 6e and Table S5 summarize the latest advancements found in the design of metal compound catalyst as sulfur host or separator modified layer in pouch cell scale, highlighting the mass percentage of catalyst to cathode, cathode size, sulfur content, areal capacity, and capacity retention in 50 cycles. Our work has promising advantages in these aspects. From the perspective of pouch cell manufacturing, designs that minimize the amount of metal additives are highly advantageous. As a demonstration, we fabricated two 12‐layer pouch cells, each with a total energy capacity of around 2 Wh, incorporating VN@hC as the electrocatalytic additive. These cells were specifically designed to power a custom‐built drone weighing 40 grams. The active material used for cathode formulation was carbon nanotubes and sulfur composite (CNT/S) in weight ratio of 1:3, with the VN@hC content of 5 wt%. The sulfur areal loading was set to be ca. 3.6 mg cm^−2^. The pouch cell assembly was conducted by first stacking the cathode, separator, and the anode, followed by prismatic winding the stacked components. Electrolyte was then added, maintaining an E/S ratio of 3.4 µL mg^−1^. All the weight of the components are summarized in Table S6. Inset picture of Figure 6f illustrates the winded cell structure of the multilayer pouch cells. Two twin multilayer pouch cells were connected in series to achieve the required power for drone lift‐off. As shown in Figure 6h, the connected pouch cells were integrated into the custom‐designed drone. A video of the successful take‐off flight is provided in the Supporting Information (Video S1). Figure S36 presents the initial charge‐discharge profile of one of the twin pouch cells, showing the high specific capacity. The calculation, detailed in Equation II in the Supporting Information, considers the mass of the whole cell. Based on this, the energy density was determined to be 233 Wh kg^−1^. Figure 6f presents the cycling performance validation, with a real photograph of the assembled cell as an inset. Following its use in powering the drone, the pouch cell was subjected to cycling at 0.1 C for up to 60 cycles to assess its stability, with an initial specific capacity of 936 mAh g^−1^. The capacity retention of the VN@hC 12‐layer pouch cell showed a promising lifespan, showing an average capacity decay per cycle of 0.48%.

Table S7 summarize the electrochemical performance metrics of TMN@C in Li–S batteries, which support the conclusion that it is primarily dictated by their morphology, where hollow structures with high surface area and confined mesoporosity enhance sulfur utilization and initial capacity, while solid architectures provide improved polysulfide confinement and structural stability, leading to superior long‐term cycling performance. The fabrication strategy presented herein opens new avenues for producing nanostructured TMNs on a large scale, as the self‐nitriding approach minimizes safety risks, keeps the procedure straightforward, and promotes environmental sustainability. Its facile scalability further offers significant practical advantages, reducing material costs and enhancing the energy density of sulfur cathodes by maximizing the utilization of active material through the electrocatalyst approach, paving the way for more efficient and cost‐effective Li–S batteries.

## 3 Conclusion

In brief, we introduce a facile method for synthesizing VN and Mo_2_N nanoparticles embedded within either hollow or dense solid carbon architectures. This approach utilizes the self‐nitriding capabilities of imidazolium‐based PILs and applies an in situ thin coating of PDA, which plays a key role in regulating ion exchange and maintaining the desired morphology of the carbon framework after thermal treatment. Subsequently, both nanostructured TMNs were evaluated within Li‐S batteries, revealing the impact of the specific TMN type combined with a hollow and confined structure on electrochemical performance. This configuration highlighted how the unique structural and catalytic properties of each TMN affect sulfur utilization, redox kinetics, and overall cyclability. As a result, VN@sC with compact configuration and moderate adsorption achieves excellent cycling stability, delivering 760 mAh g^−1^ initially and retaining 495 mAh g^−1^ after 650 cycles at 2 C in coin cells. Hollow architecture (VN@hC, Mo_2_N@hC) balances catalyst distribution and structure, requiring only 5 wt% additive while maintaining high sulfur utilization of 950 and 980 mAh g^−1^ after 50 cycles in large‐format pouch cells (7.9 × 11 cm^2^). The successful scale‐up to multilayer pouch cells highlights the practical viability of this strategy, unlocking opportunities for next‐generation lightweight, high‐energy systems tailored for drone power applications.

## Conflicts of Interest

The authors declare no conflicts of interest.

## Supporting information




**Supporting File 1**: advs74841‐sup‐0001‐SuppMat.docx.

**Supporting File 2**: advs74841‐sup‐0002‐VideoS1.mp4.

## Data Availability

The data that support the findings of this study are available from the corresponding author upon reasonable request.
