# Supplementary material for: Self‐Nitriding Nanostructured Transition Metal Nitrides in Architected‐Carbon Matrices: Unveiling Mechanisms and Advancing Performance in Lithium‐Sulfur Pouch Cells
Source: Adv Sci (Weinh). 2026 Mar 31;13(30):e21940. doi: 10.1002/advs.202521940 (PMC13248856; doi:10.1002/advs.202521940)
Supplement: Supplementary file 1 — Supporting File 1: advs74841‐sup‐0001‐SuppMat.docx. [file ADVS-13-e21940-s002.docx]

**Supporting information**

**Self-nitriding Nanostructured Transition Metal Nitrides in Architected Carbon Matrices: Unveiling Mechanisms and Advancing Performance in Lithium-Sulfur Pouch Cells**

*Yael Rodriguez Ayllon, Liqiang Lu, Dongjiu Xie, Xuefeng Pan, Teodor Jianu, Fangmu Qu, Sijia Cao, Yu Zhang,* *Roberto Félix, Marcus Bär, Nadezda V. Tarakina, Johannes Schmidt, Qingping Wu^*^, Jiayin Yuan, Yan Lu^*^.*

Y. R. Ayllon, L. Lu, D. Xie, X. Pan, S. Cao, Q. Wu, Y. Lu

Institute of Electrochemical Energy Storage, Helmholtz-Zentrum Berlin für Materialien und Energie, Hahn-Meitner-Platz 1, 14109 Berlin, Germany

**E-mail:** [yan.lu@helmholtz-berlin.de](mailto:yan.lu@helmholtz-berlin.de), [qingping.wu@helmholtz-berlin.de](mailto:qingping.wu@helmholtz-berlin.de)

Y. R. Ayllon, F. Qu, Y. Lu

Institute for Technical and Environmental Chemistry, Friedrich-Schiller-Universität Jena, Philosophenweg 7b, 07743 Jena, Germany

R. Felix, M. Bär,

Department of Interface Design, Helmholtz-Zentrum Berlin für Materialien und Energie, Albert-Einstein-Str. 15, 12489 Berlin, Germany

M. Bär,

Energy Materials In-Situ Laboratory Berlin (EMIL), Helmholtz-Zentrum Berlin für Materialien und Energie, Albert-Einstein-Str. 15, 12489 Berlin, Germany

Department of X-ray Spectroscopy at Interfaces of Thin Films, Helmholtz Institute Erlangen-Nürnberg for Renewable Energy (HIERN), Albert-Einstein-Str. 15, 12489 Berlin, Germany

Department of Chemistry and Pharmacy, Friedrich-Alexander-Universität Erlangen-Nürnberg (FAU), Egerlandstr. 3, 91058 Erlangen, Germany

T. Jianu, N. V. Tarakina

Department of Colloid Chemistry, Max Planck Institute of Colloids and Interfaces, 14476 Potsdam, Germany

Y. Zhang, J. Yuan

Department of Materials and Environmental Chemistry, Stockholm University, Stockholm 10691, Sweden

J. Schmidt

Department of Chemistry, Functional Materials, Technical University Berlin, 10623 Berlin, Germany

Q. Wu

Chongqing Institute of Green and Intelligent Technology, Chinese Academy of Sciences, Chongqing 400714, China.

Y. Lu

Helmholtz Institute for Polymers in Energy Applications (HIPOLE Jena), Lessingstr. 12-14, 07743 Jena, Germany

**Materials**

1-vinylimidazole (≥ 99%), 1-bromodecane (98%), butylated hydroxytoluene (BHT) (99%), and dopamine hydrochloride (DA) (≥ 99%) were purchased from Sigma-Aldrich. 2,2' azobis[2-methyl-N-(2-hydroxyethyl) propionamide] (VA086) (99%) was purchased from Fujifilm. Ammonium orthomolybdate ((NH_4_)_2_MoO_4_) (99.5%) and ammonium metavanadate (99.5%) were purchased from Acros organics. Tris(hydroxymethyl) aminomethane (Tris) (99.9%) and methanol (analytical grade) (99.9%) were purchased from Carl-Roth. Deionized water (DI-water), used for all aqueous solutions and dispersion, was obtained from an ion exchange (Millipore Milli-Q) system. The materials for electrochemical testing were obtained as follows: sublimed sulfur (99.5%) was purchased from Alfa-Aesar. Carbon disulfide (CS_2_) (99.9%), 1-methyl-2-pyrrolidinone (NMP) (99%), lithium bis-trifluoromethanesulfonimide (LiTFSI) (99%), lithium nitrate (LiNO_3_) (≥ 99.9%), 1, 3-dioxolane (DME) (99.5%), 1,2-dimethoxyethane (DOL) (99.5%), and lithium disulfide (Li_2_S) (99.9%) were purchased from Sigma-Aldrich. Polyvinylidene fluoride (PVDF) (99.5%) was purchased from MTI corporation. Carbon Ketjen black EC-300J (KB) and carbon nanotubes (CNTs) were purchased from Lion Specialty chemicals. Acrylonitrile Multi-Copolymer Binder (LA133) was purchased from Indigo Co. Celgard 2700® membrane was purchased from Celgard LLC. Lithium foil (99.9%) was obtained from China Energy Lithium Co. All the chemicals were used as received without further purification.

**Synthesis of Ionic Liquid Monomer and PIL spheres**

In a typical run^[S1-2]^, 50 mg of BHT was dissolved in 10 mL of methanol, followed by the addition of 0.1 mol of 1-vinylimidazole, and 0.1 mol of 1-bromodecane. The mixture was stirred using a magnetic bar at 60 °C for 15 h. The system was repeatedly added to *ca.* 300 mL of diethyl ether (DEE) for precipitation of the IL monomer ILM-C10Br. The solid powder at the bottom was collected and dried in a high vacuum oven overnight at room temperature. The polymerization of the as-synthesized 3-*n*-decyl-1-vinylimidazolium bromide (ILM-C10Br) was conducted as follows. 1.2 g of ILM-C10Br was dissolved in 100 mL of deionized water (final concentration of 12 mg mL^-1^). Subsequently, 150 mg of initiator VA086 was added under sonication. Then, the air inside the system was purged away and refilled with nitrogen gas three times using a schlenk line. The polymerization was conducted at 75 °C for 20 h. After that, a stable colloidal dispersion was observed, and was collected in a dialysis bag for exhaustive dialysis against DI water. The water was replaced until the conductivity was below 2.5 µS cm^-1^. The synthesis of the dense PIL spheres in a colloidal dispersion followed exactly the same procedure, except the ILM-C10Br concentration at 24 mg mL^-1^.

**Synthesis of nanostructured Mo_2_N and VN embedded in carbon spheres**

In a typical run for both hollow and porous Mo_2_N@C spheres, 38.4 mg mL^-1^ (192 mg in 5 mL) of ammonium orthomolybdate was dissolved in deionized water, followed by the addition of 4 mg mL^-1^ (20 mg) of DA under pulse sonication at *ca.* 10 °C until a homogeneous burgundy solution was observed. In the meantime, the PILs concentration was adjusted with deionized water to 2.850 mg mL^-1^ (100 mg in 35 mL). Subsequently, the pH of the PIL dispersion was adjusted to *ca.* 10 by adding 10 mM (48.5 mg) of Tris under pulse sonication at *ca.* 10 °C. The freshly prepared solution containing dopamine and molybdenum ions was introduced dropwise to the PILs dispersion under sonication at *ca.* 10 °C. The resulting mixture underwent stirring at 500 RPM in an ice bath for 2 h and then transferred into an oil bath at 80 °C (heating rate of 10 °C min^-1^) with continuous stirring at 500 RPM for an additional 20 h, leading to the transformation of the dispersion into a black hue.

To synthesize both hollow and porous VN@C spheres, we employed the same method with variations made solely to the quantities of materials used. A 5.7 mg mL^-1^ (114 mg in 20 mL) of ammonium metavanadate was dissolved in deionized water followed by the addition of 1 mg mL^-1^ (20 mg) of DA. The PILs concentration was adjusted with deionized water to 5 mg mL^-1^ (100 mg in 20 mL).

The resulting products were gathered through centrifugation at 9000 RPM for 15 minutes and washed twice against DI-water. The systems were redispersed in *ca.*10 mL of water and freeze-dried until complete removal of the water was achieved (around 7 h). The obtained powders were placed into a ceramic crucible and subjected to thermal treatment using a tube furnace at various temperatures ranging from 600 to 1000 °C under a continuous Argon flow (keeping the pressure at 0.2 bar) with a heating rate of 3 °C min^-1^. After naturally cooling down to room temperature (normally 20 h) in a fresh argon flow, the samples were taken out and stored in a glove box (H_2_O content < 0.5 ppm and O_2_ content < 1 ppm).

**Preparation of hollow carbon spheres and bulk N-doped carbon**

The hollow carbon spheres used for electrochemical comparison were obtained by etching off the Mo_2_N nanoparticles prepared at 600 °C from the carbon template. This was achieved by immersing the sample in a 1 M HCl solution for 20 h, followed by thorough washing with ethanol and water. The etching procedure was iterated several times until a colorless supernatant was observed. The sample was dried and treated at 600 °C in an argon atmosphere for 1 h and heating rate of 3 °C min^-1^. The bulk N-doped carbon was obtained under the same synthesis conditions without any Mo/V-based salt, and then treated at 600 °C in an argon atmosphere for 3 h and heating rate of 3 °C min^-1^.

**Static adsorption of lithium polysulfides**

The lithium polysulfide solution (Li_2_S_6_) was formulated by dissolving sulfur and Li_2_S in a stoichiometric ratio of 5:1 (molar) in a mixed solvent of DOL/DME (1:1, v/v). The solution was subjected to vigorous stirring at 80 °C for 48 h in an argon atmosphere. Subsequently, an equal mass of 30 mg of sample was introduced into 3 mL of LiPSs solution (2 mM) for each sample. Following an undisturbed aging period of 3 h in a glovebox, the supernatant liquid was collected and sealed in a quartz cuvette for UV–vis spectroscopy analysis.

**Sulfur cathode formulation and coin cell assembly**

All the host materials and sublimed sulfur were initially mixed in a mass ratio of 3:7 by grinding with a mortar and pestle, then CS_2_ was added to completely immerse the mixture. Following complete solvent evaporation, the mixture was grinded and sealed in an autoclave under argon and heated at 155 °C for 12 h. Subsequently, the electrode sheet was prepared by casting a NMP-based slurry consisting of the as-prepared sulfur composite, Ketjen black (KB), and PVDF in a 7:2:1 mass ratio onto a battery-grade Al foil using a doctor blade. The areal sulfur loading of the electrode sheet was set to approximately 2 mg cm^–2^. After drying at 45 °C under vacuum overnight, the electrode sheet was calendared once at room temperature with rolling gap of 0.15 mm. The sheet was then cut into several wafers with a diameter of 14 mm.

The cell assembly was conducted in an Argon-filled glove box (UNIlab plus, M. BRAUN) with H_2_O content < 0.5 ppm and O_2_ content < 0.5 ppm. The coin cells (CR2032 type) were assembled by using the as-prepared sulfur cathode, Celgard 2700 ® membrane as a separator,1 M LiTFSI solution with 2 wt% LiNO_3_ in the mixed solution of DME: DOL (v/v: 1.1) as the electrolyte, and Li foil disk (14 mm in diameter) as the anode. The electrolyte-to-sulfur ratio (E/S) was set to *ca.* 13 µL mg^-1^. The assembled coin cells were aged under open circuit potential for 10 h at room temperature to allow the electrolyte to wet the electrodes. The current density at 1 C was considered to correspond to the theoretical capacity of sulfur (1675 mA g^–1^). The specific capacity is then determined by calculating the mass of sulfur content in each electrode.

Galvanostatic discharging–charging experiments were performed using a Neware battery testing system (CT-4008-5 V10 mA) at room temperature within the electrochemical window of 1.7–2.8 V vs Li/Li^+^. Electrochemical impedance spectroscopy (EIS) data were acquired using a GAMRY Interface 1000 over a frequency range from 100 kHz to 0.01 Hz at open circuit potential. Cyclic voltammetry (CV) curves were recorded employing a Biologic VMP3 electrochemical workstation with a scanning rate of 0.1 mV s^–1^ within the electrochemical window of 1.7–2.8 V vs Li/Li^+^.

**Sulfur cathode with TMN electrocatalysts and Pouch cell assembly**

Sublimed sulfur and Ketjen black (KB) were mixed in a mass ratio of 85:15, followed by a molten sulfur infusion method at 155 ^o^C for 12 hours. The as-prepared KBS composite was then mixed with the transition metal nitride additive and Super P conductive carbon in a mass ratio of 90:5:5. The slurry formulation was prepared by mixing the above composite with LA133 binder (dissolved in water) in a mass ratio of 95:5. The electrode sheet was prepared by casting slurry onto carbon-coated Al foil using a doctor blade. After drying at 45 °C under vacuum overnight, the electrode sheet was calendared and cut according to the pouch cell size (4.4 cm*5.7 cm and 7.9 cm*11.0 cm). The electrodes were further dried at 60 °C for 12 hours before being transferred to glovebox. The areal sulfur loading of the electrode sheet was set in a range of 3.3-3.6 mg cm^–2^. The cathodes without catalysts were prepared in a similar process but without adding catalysts during the materials mixing step.

The pouch cell assembly was conducted in an Argon-filled glove box (UNIlab plus, M. BRAUN) with H_2_O content < 0.5 ppm and O_2_ content < 0.5 ppm. The assembly was done using the as-designed sulfur cathode, Celgard 2700 ® membrane as a separator, 1 M LiTFSI solution with 2 wt% LiNO_3_ in the mixed solution of DME: DOL (v/v: 1.1) as the electrolyte, and Li foil coated cupper foil as the anode. The electrolyte-to-sulfur ratio (E/S) was set to *ca.* 5 or 10 µL mg^-1^. The electrolyte was injected from the top and sealed under vacuum.

Galvanostatic discharging–charging experiments were performed using an Arbin Instruments model LBT21084 at room temperature within the electrochemical window of 1.7–2.8 V vs Li/Li^+^.

For the multilayer pouch cells for drone, the cathode material was prepared by mixing sulfur, carbon nanotubes (CNTs) with a mass ratio of 3:1, followed by adding 5 wt% VN catalyst. The slurry containing cathode material and LA133 binder with a ratio of 95:5 was casted onto Al foil, followed by drying, calendering and cutting into strips with the size of 4 cm* 30 cm. The pouch cells were then prepared by winding the stacked cathodes, separator and anodes, electrolyte injection and vacuum sealing.

**Electrode formulation for catalytic activity test and Li_2_S precipitation**

The pristine host material was combined with Ketjen black and PVDF in a mass ratio of 8:1:1, facilitated by NMP dispersion in a mortar. The resulting slurry was meticulously mixed with a pestle until achieving a homogeneous dispersion. Subsequently, the mixture was applied onto commercial carbon paper using a conventional casting method. The coating area was carefully defined to ensure the deposition of approximately 1 mg cm^–2^ of the host material. After drying at 45 °C under vacuum overnight, the electrode was cut into wafers with a diameter of 14 mm.

**Symmetrical cell testing towards lithium polysulfides**

Two identical electrodes with the same mass were used as working and counter electrode, respectively. The CR2032 coin cells were assembled in an Argon-filled glove box. Celgard 2700 ® membrane was used as a separator. 40 μL of Li_2_S_6_ (2.5 M, based on sulfur content) solution with 1.0 M LiTFSI in the solvent of DOL and DME (1:1, v/v) was used as catholyte. The symmetric battery was measured a Biologic VMP3 electrochemical workstation at a scan rate of 10 mV s^–1^ in a potential window from −0.8 to 0.8 V.

**Kinetics of Li_2_S Precipitation.**

The CR2032 coin cells were assembled in an Argon-filled glove box, using a pristine host material cathode loaded with 20 μL catholyte consisting of Li_2_S_8_ (0.25 M) solution with 1 M LiTFSI in the mixture solvent of DOL and DME (1:1, v/v), Celgard 2700 ® membrane was used as a separator, 20 μL of 1 M LiTFSI in DOL and DME (1:1, v/v) as anolyte, and Li foil disk (14 mm in diameter) as the anode. After assembly, the cells were aged for 2 h and then discharged galvanostatically at a current density of 0.1 C to 2.16 V vs. Li/Li^+^ and then discharged potentiostatically at 2.05 V vs Li/Li^+^ for Li_2_S nucleation and growth. The potentiostat discharge was set over 65000 s.

**Density functional theory (DFT) for adsorption of Li_2_S_6_ on Mo_2_N (111) or VN (200)**

Density Functional Theory (DFT) calculations were performed using the Vienna Ab-initio Simulation Package (VASP)^[S3]^ with the projector augmented wave (PAW) method.^[S4]^ The exchange-correlation functional was treated using the Perdew-Burke-Ernzerhof (PBE)^[S5]^ functional, in combination with the DFT-D3 dispersion correction.^[S6]^ The plane-wave cutoff energy was set to 450 eV. In model construction, a slab model of γ-Mo_2_N (111) 2×2×1 supercell (Mo_38_N_38_) was built based on the γ-Mo_2_N bulk structure. A VN (200) 4×3×1 supercell was constructed based on the cubic VN bulk structure. For both slab models, the thickness along the c-direction was set to 30 Å to minimize interactions between periodic images. For structural optimization: The lattice parameters of BCC Li_2_ bulk were optimized using a Monkhorst-Pack^[S7]^ k-point grid of 7×7×7. The lattice parameters of S_128_ bulk were optimized using a 2×2×1 k-point grid. The γ-Mo_2_N (111) 2×2×1 supercell lattice was optimized using a 2×2×1 k-point grid. The lattice parameters of VN bulk were optimized using a 6×6×6 k-point grid. The geometry of the VN (200) slab was optimized using a 1×2×1 k-point grid, with some atoms fixed to simulate surface constraints. Self-consistent calculations employed an energy convergence threshold of 10^-5^ eV. Geometries and lattice constants were optimized until the maximum force on any atom was less than 0.02 eV Å^-1^. The binding energies *E_b_* of Li_2_S_6_ species with Mo_2_N (111) and VN (200) sulfur hosts were calculated as:

$$E_{b}=E_{{Li}_{2}S_{6}}+E_{host}-E_{{Li}_{2}S_{6}+host}$$

where$E_{{Li}_{2}S_{6}+host}$, $E_{host}$, and $E_{{Li}_{2}S_{6}}$ represent the total energy of Li_2_S_6_ cluster with Mo_2_N (111) or VN (200), total energy of Mo_2_N (111) or VN (200), and total energy of Li_2_S_6_ cluster, respectively.

Spin polarization was included to describe the magnetic properties of the slab models. The density of states (DOS) of the slab models was calculated using the GGA-PBE functional and processed through the VASPKIT interface.^[S8]^

The elementary steps of the sulfur reduction reaction (SRR) on the slab models were then analyzed as follows:

*S_8_ + Li_2_(s) → *Li_2_S_8_

*Li_2_S_8_ → *Li_2_S_6_ + 1/64S_128_(s)

*Li_2_S_6_ → *Li_2_S_4_ + 1/64S_128_(s)

*Li_2_S_4_ → *Li_2_S_2_ + 1/64S_128_(s)

*Li_2_S_2_ → *Li_2_S + 1/128S_128_(s)

In the reaction pathways, * denotes the bare surface of the slab model, whereas *i represents the slab with intermediate *i adsorbed on the surface. The Gibbs free energy of i was calculated as*

$$G=E+G(T),$$

Where $E$is the DFT total energy of *i* and $G(T)$is the thermal correction. The thermal correction $G(T)$includes two contributions: the zero-point energy *(ZPE)* and the entropic term, expressed as the product of temperature and entropy *(–*$TS$*).* The thermal correction for each i was obtained using the VASPKIT interface. All calculations were performed at 298.15 K.

**Material characterization.**

To determine the morphology of the particles, a transmission electron microscope (TEM JEOL JEM-2100 with LaB6 cathode) was used. The TEM was operated at an acceleration voltage of 200 kV. Cryo-TEM specimens were prepared by dropping 4 μL of a dispersion sample to Lacey carbon-coated copper TEM grids (200 mesh, Science Services) and plunge-frozen into liquid ethane with an FEI vitrobot Mark IV set at 4 °C and 95% humidity. Vitrified grids were transferred to the cryogenic transfer holder (Gatan 914, Gatan, Munich, Germany) and placed in the microscope. Imaging was carried out at temperatures around 90 K. STEM measurements were performed using a double-Cs corrected JEOL JEM- ARM200F (S)TEM, operated at 200 kV using a probe semi convergence angle of 25 mrad, equipped with a cold-field emission gun, an energy dispersive X-ray detector and a Gatan Imaging Filter (GIF) Quantum. All samples were prepared on standard TEM Cu or Au grids with lacey carbon support. ADF-STEM images were recorded within an angular collection semi-angle range from 50 mrad to 180 mrad. BF-STEM images were taken from scattering signal to a maximal collection semi-angle of 3.8 mrad. EELS data were acquired using a dispersion of 0.25 eV/channel. X-ray diffraction (XRD) patterns were collected in a Bruker D8 diffractometer in the locked coupled mode (2θ ranging from 10° to 90°) with Cu Kα1 radiation (wavelength of 1.5406 Å). X-ray photoelectron spectroscopy (XPS) was performed in ESCA-Lab-220i-XL (Thermo Fisher Scientific) with Al Kα sources (hν = 1486.6 eV) to investigate the surface composition and chemical state of the materials. The specific surface area and the pore size distribution were obtained by Nitrogen adsorption/desorption using Quantachrome Autosorb-1 systems performed at 77 K. Brunauer-Emmett-Teller (BET) method was used to calculate the specific surface area based on a multipoint analysis. The TMN content, determined via thermal oxidation, and the validation of the self-nitriding mechanism were investigated using thermogravimetric analysis (TGA) performed on a Netzsch TG 209 F1 Iris instrument coupled to a QMS 403 D Aëolos mass spectrometer, under an argon or synthetic air atmosphere. The analysis was conducted at a heating rate of 10 °C min⁻¹, scanning from room temperature to700 °C. Elemental analysis of PILs was done in a Eurovector EA 3000, Co. Leintech. The Raman spectra were collected on an XPLORA plus Raman microscope with a 532 nm laser. The SEM morphology of the electrodes was determined using a scanning electron microscope Zeiss Crossbeam 340. Operando Raman measurements were performed in a DXR3 Raman Microscope. The electrodes were assembled in an operando Raman cell with the electrode surface oriented toward the optical glass window. The Raman laser (wavelength of 532 nm) was pointed into the active material while recording. Mo L_3_- and V K-edge X-ray absorption spectroscopy (XAS) measurements were conducted using the high kinetic energy electron (HiKE) end-station ^[S9]^ located at the KMC-1 beamline ^[S10]^ of BESSY II, operated by HZB. Monochromatization of the incoming X-rays for the Mo L_3_-edge XAS measurements (i.e., energy range: 2490 – 2570 eV) was achieved using the Si(111) crystal pair of the double-crystal monochromator (DCM); while for the V K-edge XAS measurements (i.e., energy range: 5450 – 5550 eV), the Si(311) crystal pair of the DCM was employed. Spectra were acquired in partial fluorescence yield (PFY) mode, with a Bruker XFlash 4010 silicon drift detector (SDD). Photon energy calibration was conducted by hard X-ray photoelectron spectroscopy (HAXPES) measurements of a clean Au foil. The low and high energy limits of the selected XAS energy ranges were used as excitation energy during calibration and the binding energy of the Au 4f_7/2_ core level was set to 84.0 eV. Prior to XAS normalization, a linear background was fitted and subtracted from each spectrum.


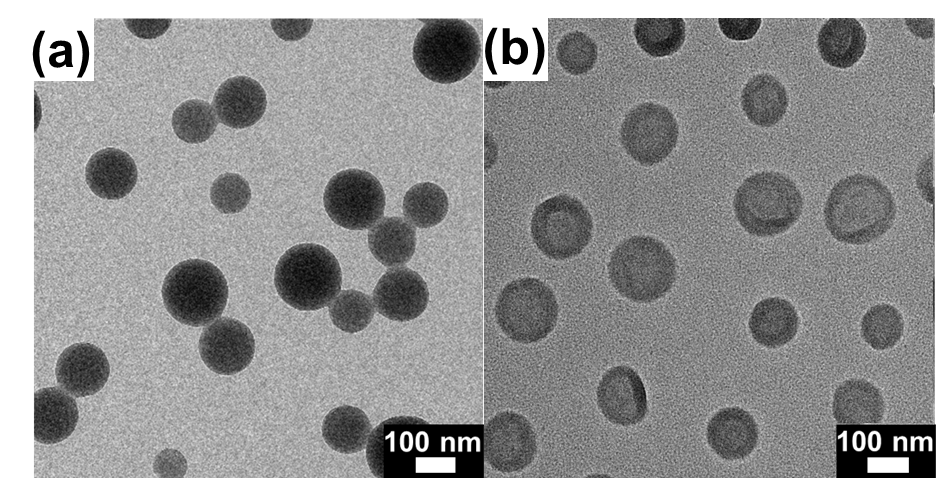


**Fig. S1**. TEM image of dense onion-like PIL spheres (a), and hollow PIL spheres (b).


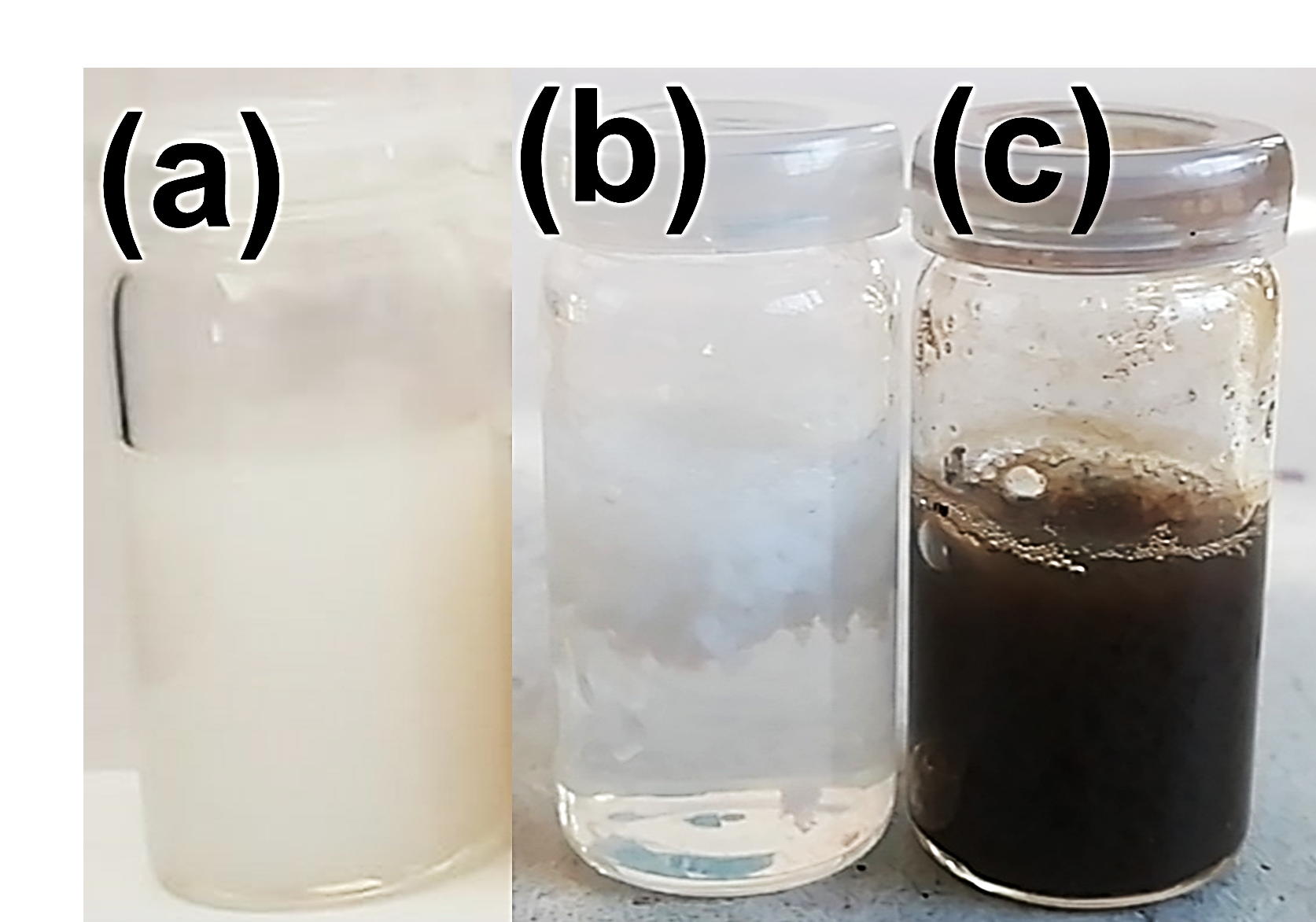


**Fig. S2**. Photograph of PILs colloidal dispersion. Pure PIL dispersion (a), PIL dispersion after direct addition of (NH_4_)_2_MoO_4_ (b), and TM-PDA@PIL dispersion after one-pot synthesis (c).


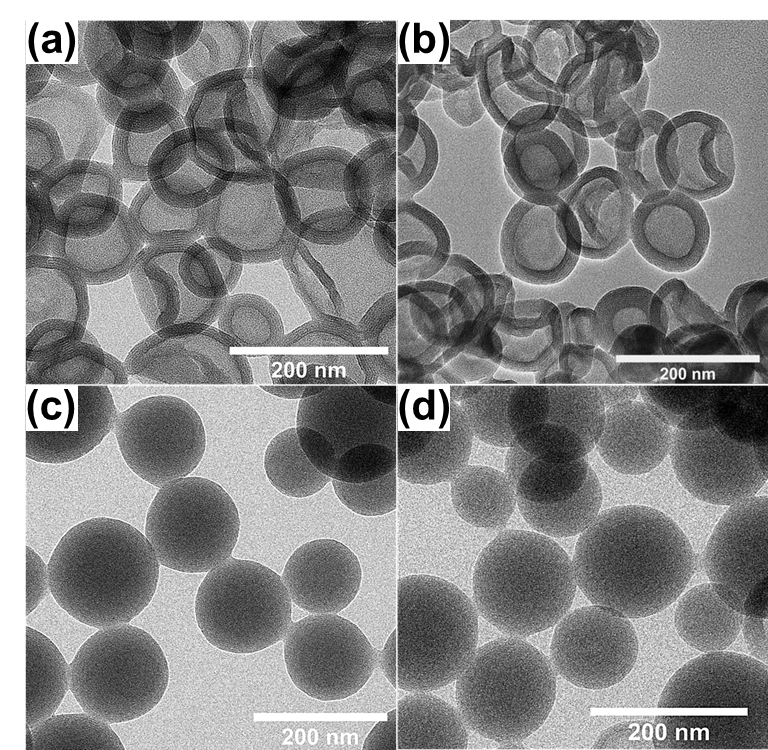


**Fig. S3**. TEM images of TM-PDA@PILs with different morphology. (NH_4_)_2_MoO_4_ (a,c). NH_4_VO_3_ (b,d).


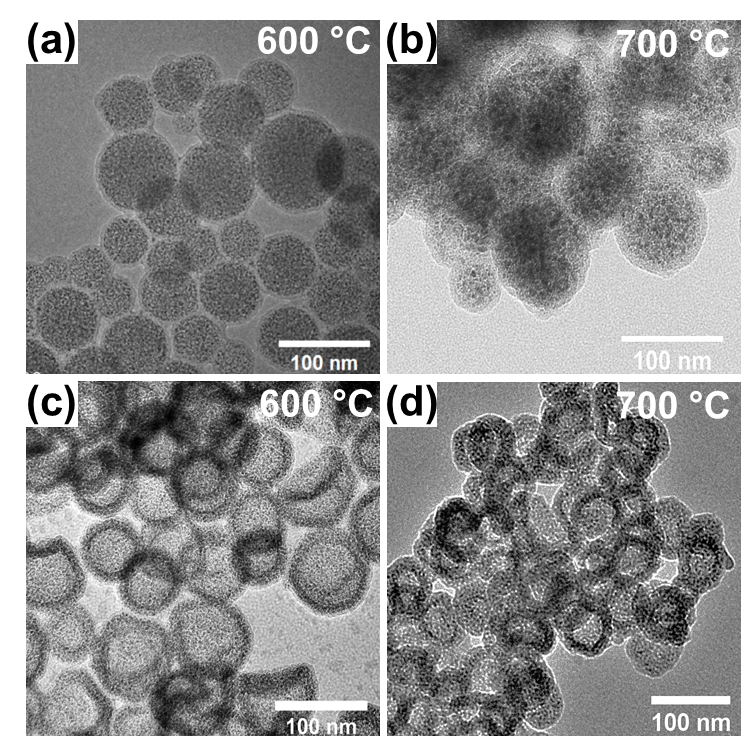


**Fig. S4.** TEM images of TMNs@C with different architectures. Mo_2_N@C (a,c), VN@C (b,d).


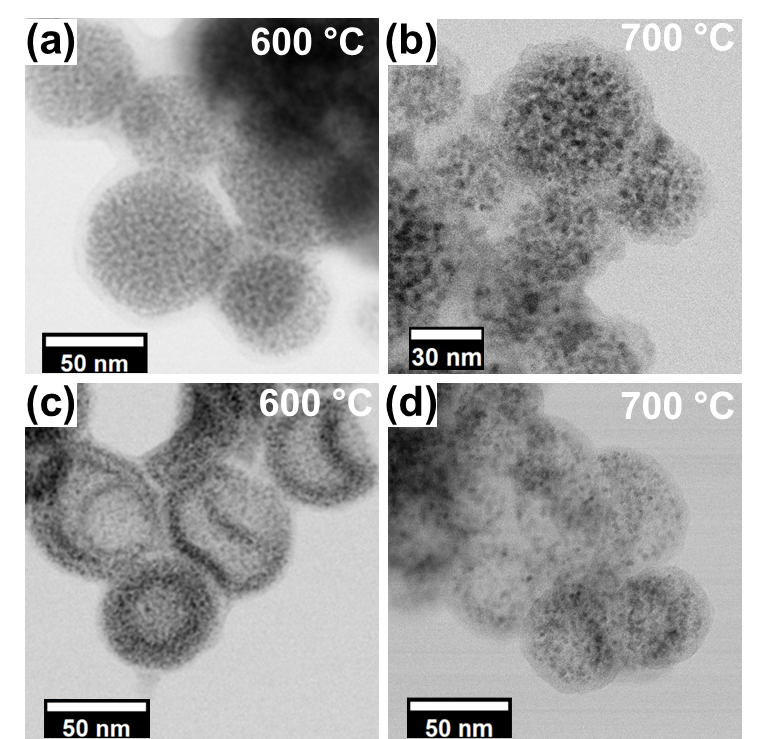


**Fig. S5**. BF-STEM images of TMNs@C with different architectures. Mo_2_N@C (a,c), VN@C (b,d).


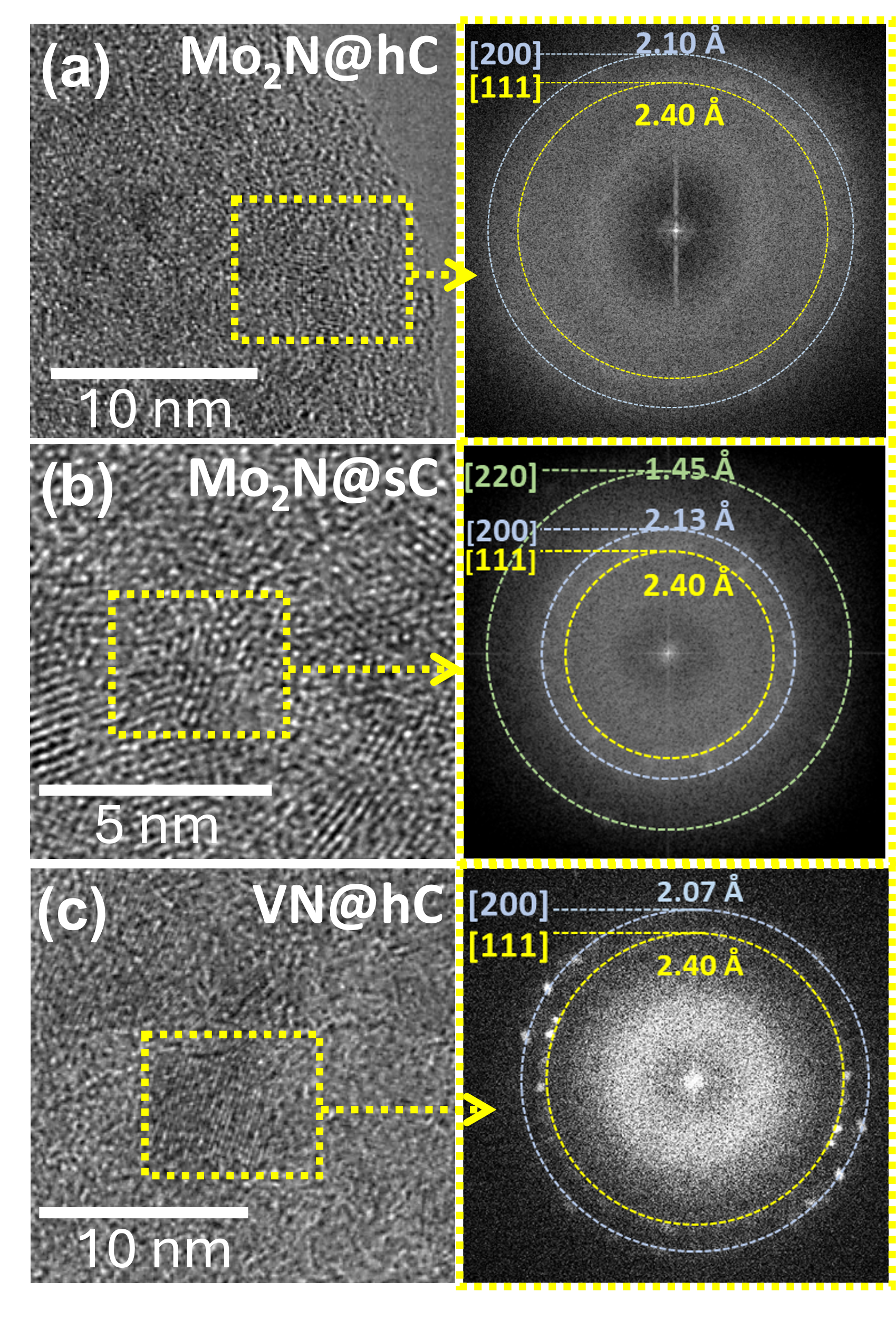


**Fig. S6**. HRTEM images of a randomly selected spot of TMNs@C and its FFT pattern. Mo_2_N@hC (a), Mo_2_N@sC (b), VN@hC (c).


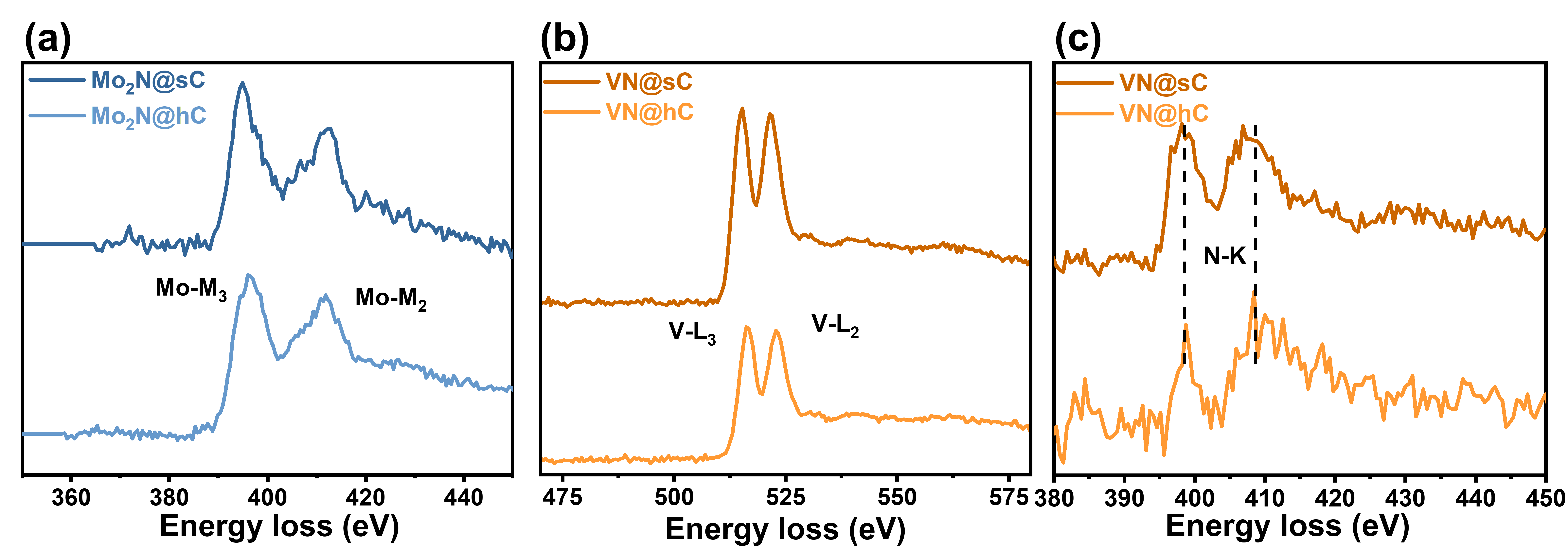


**Fig. S7**. EELS for Mo in Mo_2_N (a), EELS for V in VN (b), EELS for N in VN (c).


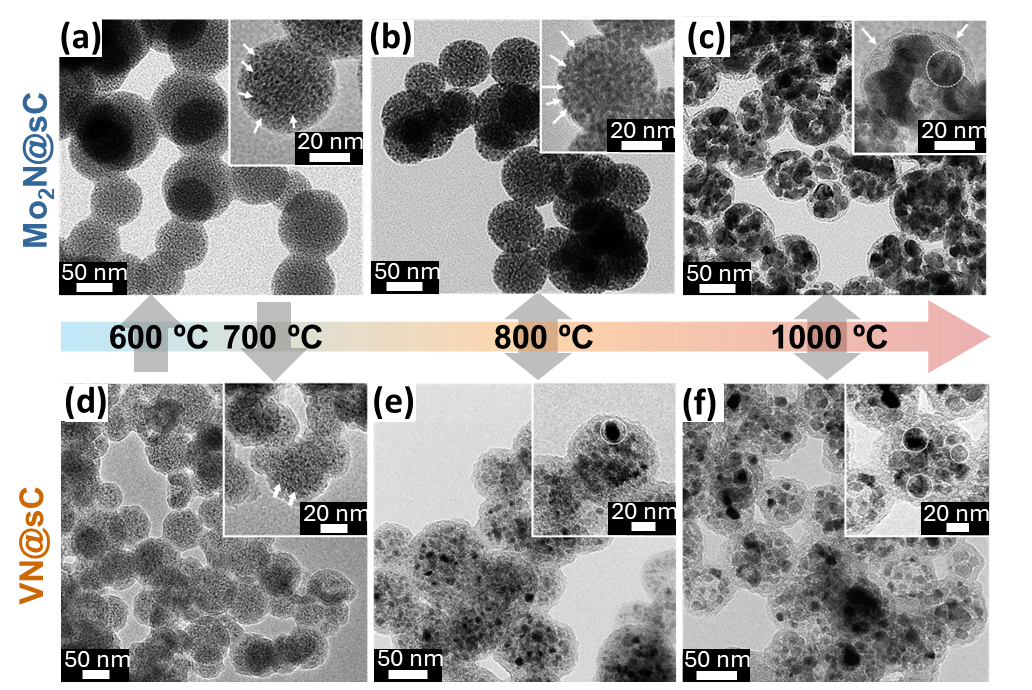


**Fig. S8**. Morphological evolution of TMNs@sC acquired at different temperatures. Mo_2_N@sC at 600, 800, and 1000 °C (a-c), VN@sC at 700, 800, and 1000 °C (d-f).

**Fig. S9.** TGA–MS evidence for the self-nitriding mechanism of V-PDA@PILs during thermal decomposition.

**Fig. S10.** Progressive compounds evolution of V-PDA@PILs during thermal decomposition.





**Fig. S11**. XRD of TMNs@hC (a). XRD patterns comparison of TMNs@sC acquired at different temperatures (600-1000 °C). Mo_2_N@sC (a), VN@sC (b).





**Fig. S12**. TGA collected under synthetic air(b). XRD patterns of waste product from the TGA of TMNs@C. Mo_2_N@C (a), VN@C (b).





**Fig. S13**. N₂ adsorption–desorption isotherms of TMNs@hC and its corresponding pore size distribution (a-b).

**Fig. S14**. Raman spectra of TMNs@hC.





**Fig. S15**. XPS spectra of bulk carbon from bare PDA@PILs. C 1s (a), N 1s (b).





**Fig. S16**. XPS spectra of VN@hC, V 2p (a), N 1s (b), C 1s (c). XPS scans of Mo_2_N@hC, Mo 3d (d), N 1s (e), C 1s (f).





**Fig. S17**. XPS spectra on C 1s for VN@sC acquired at 700 °C (a), 800 °C (b), and 1000 °C (c).





**Fig. S18**. XPS spectra on C 1s for Mo_2_N@sC acquired at 600 °C (a), 800 °C (b), and 1000 °C (c).


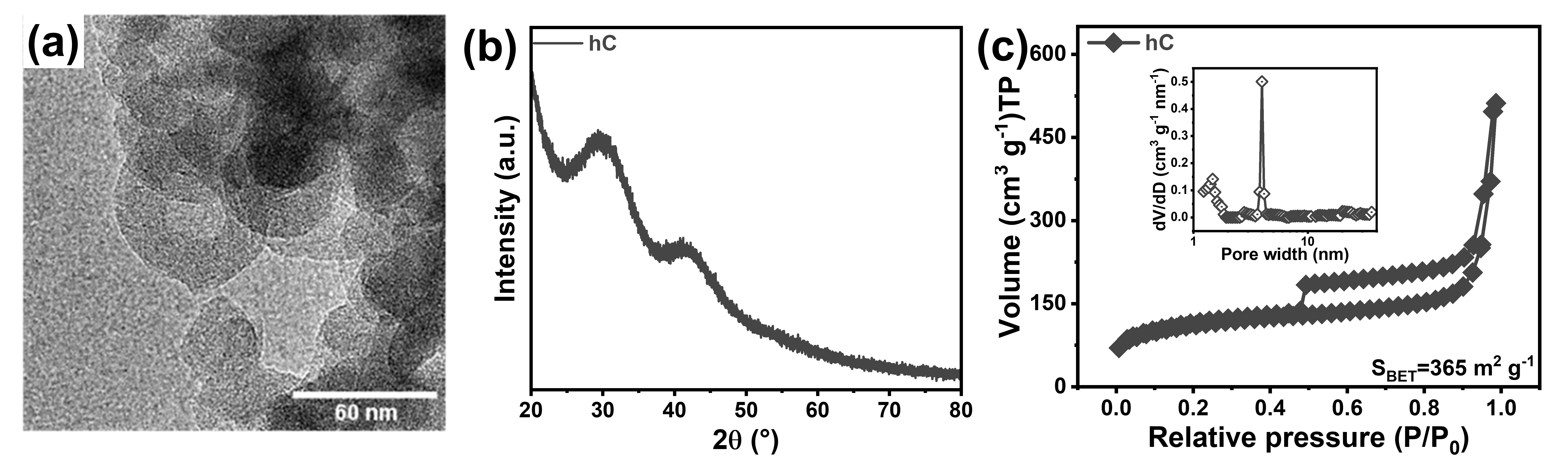


**Fig. S19**. Hollow carbon (hC) after etching. TEM image (a), XRD patterns (b), N_2_ adsorption-desorption isotherms and its corresponding pore size distribution.


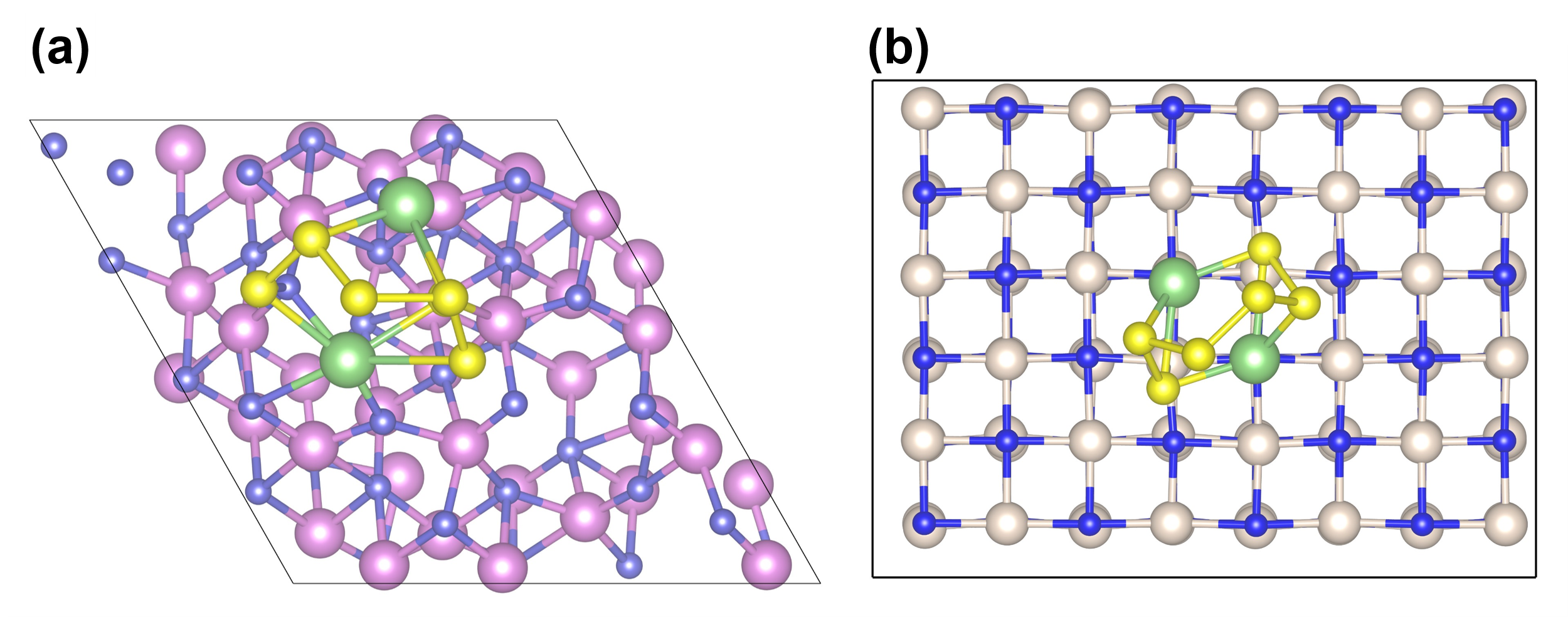


**Fig. S20**. Top-view of DFT calculations of Li_2_S_6_ on Mo_2_N (111) (a), and VN (200) (b).


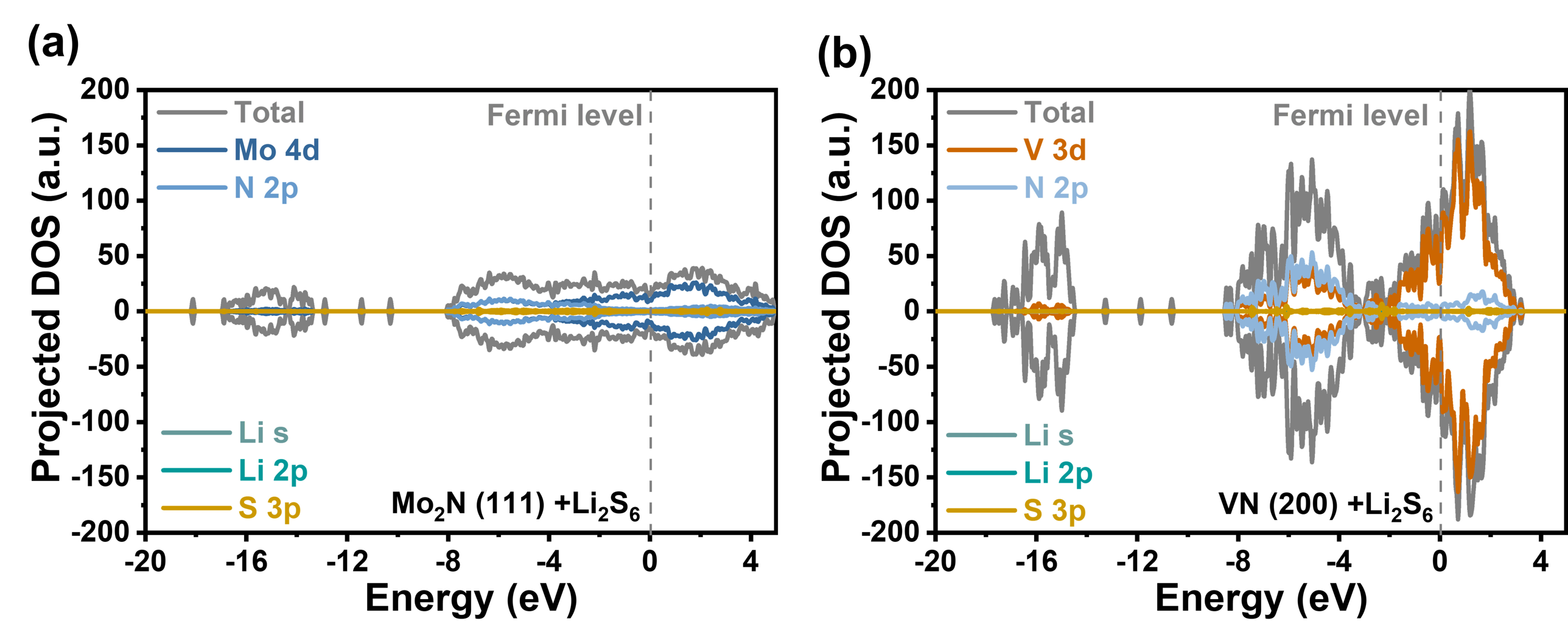


**Fig. S21**. Projected density of states (PDOS) of Li_2_S_6_ adsorbed on (a) Mo_2_N (111) and (b) VN (200).


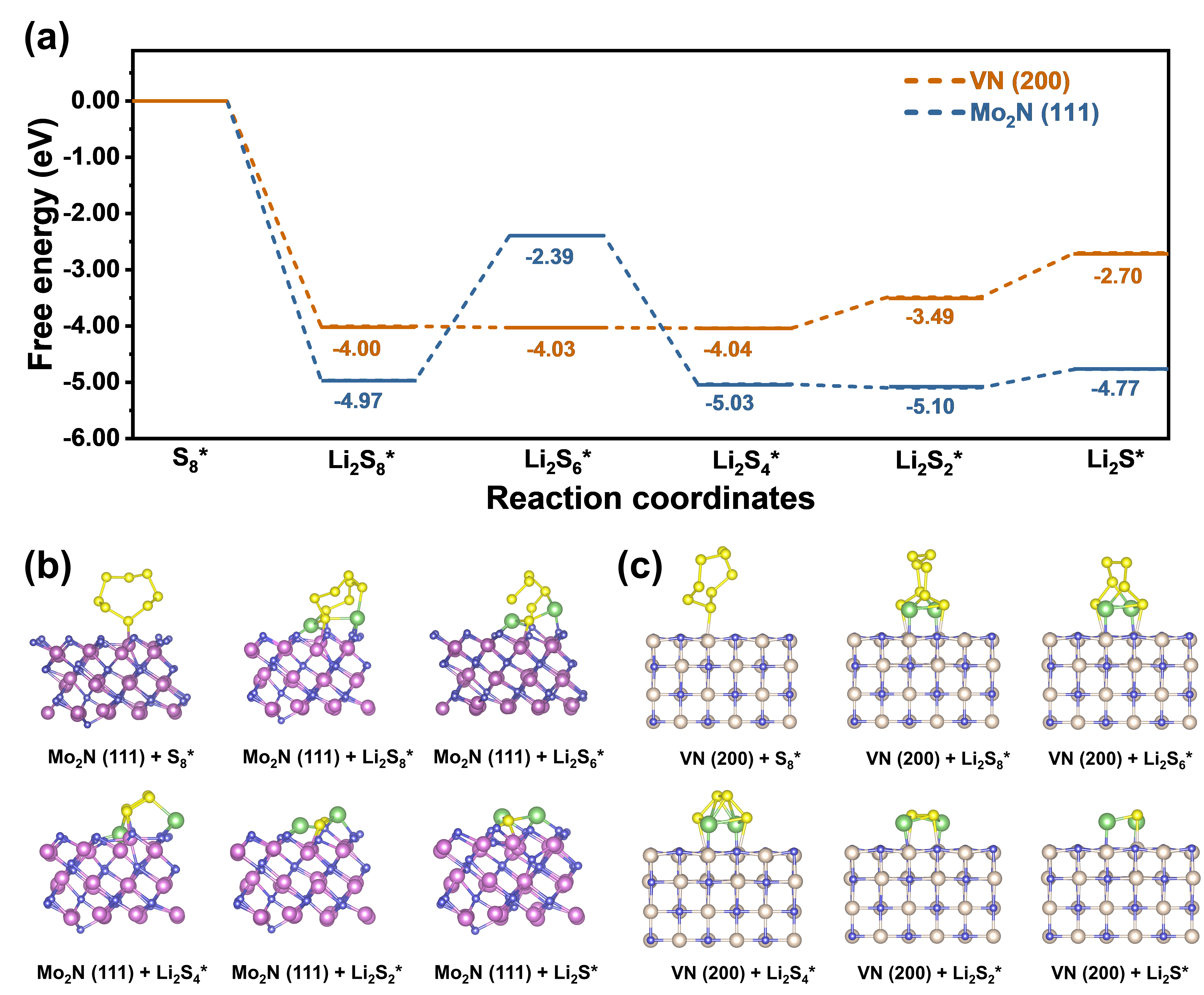


**Fig. S22.** DFT calculated sulfur reduction on TMN surfaces. (a) Gibbs free energy profiles for the stepwise conversion of S₈* to Li₂S* via Li₂S₈*, Li₂S₆*, Li₂S₄* and Li₂S₂* intermediates on Mo₂N (111) and VN (200). (b) Optimized adsorption configurations of S₈*, Li₂S₈*, Li₂S₆*, Li₂S₄*, Li₂S₂* and Li₂S* on Mo₂N (111). (c) Corresponding configurations on VN (200). Purple, gray, blue , yellow and green spheres denote Mo, V, N, S and Li atoms, respectively.


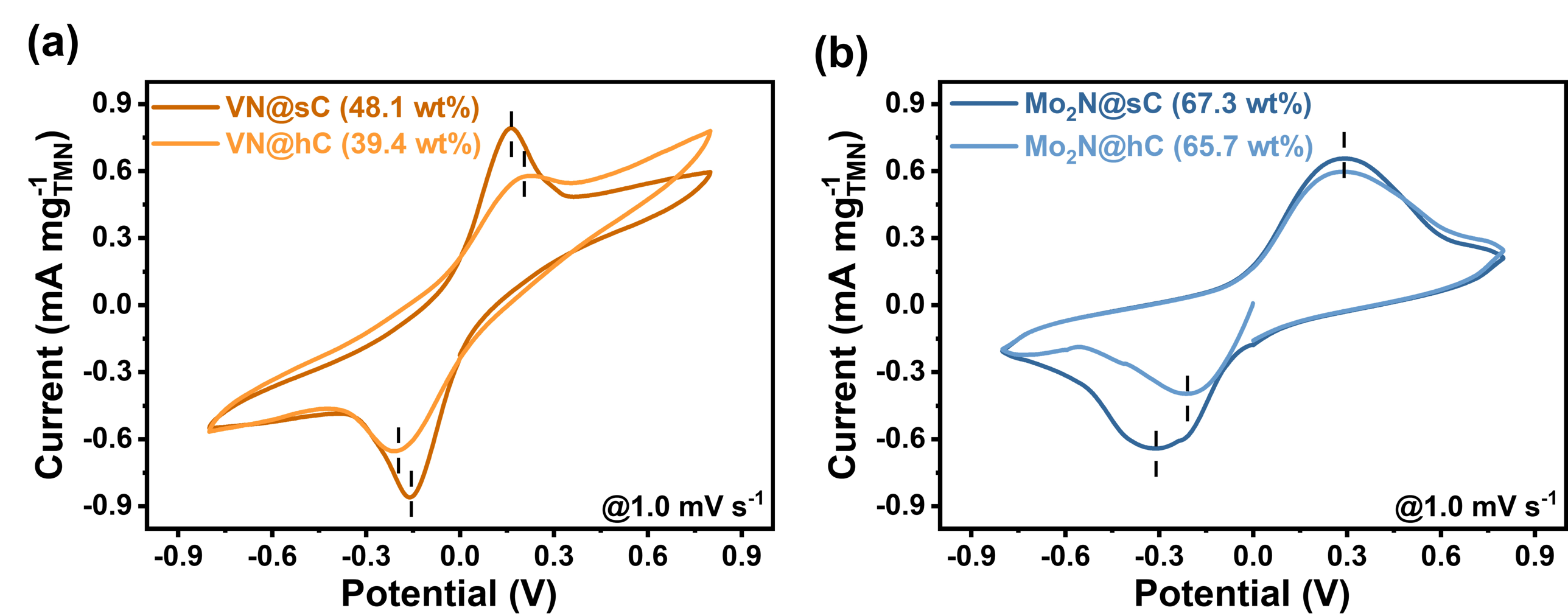


**Fig. S23.** TMN-mass-normalized cyclic voltammetry curves of symmetrical cells at a scan rate of 1 mV s⁻¹ of VN@C (a) and Mo₂N@C (b).

**Fig. S24.** Sulfur content validation via TGA in argon atmosphere of the host materials.

**Fig. S25.** Overpotential shift analysis of CV curves of host materials obtained at 0.1 mV s^-1^.


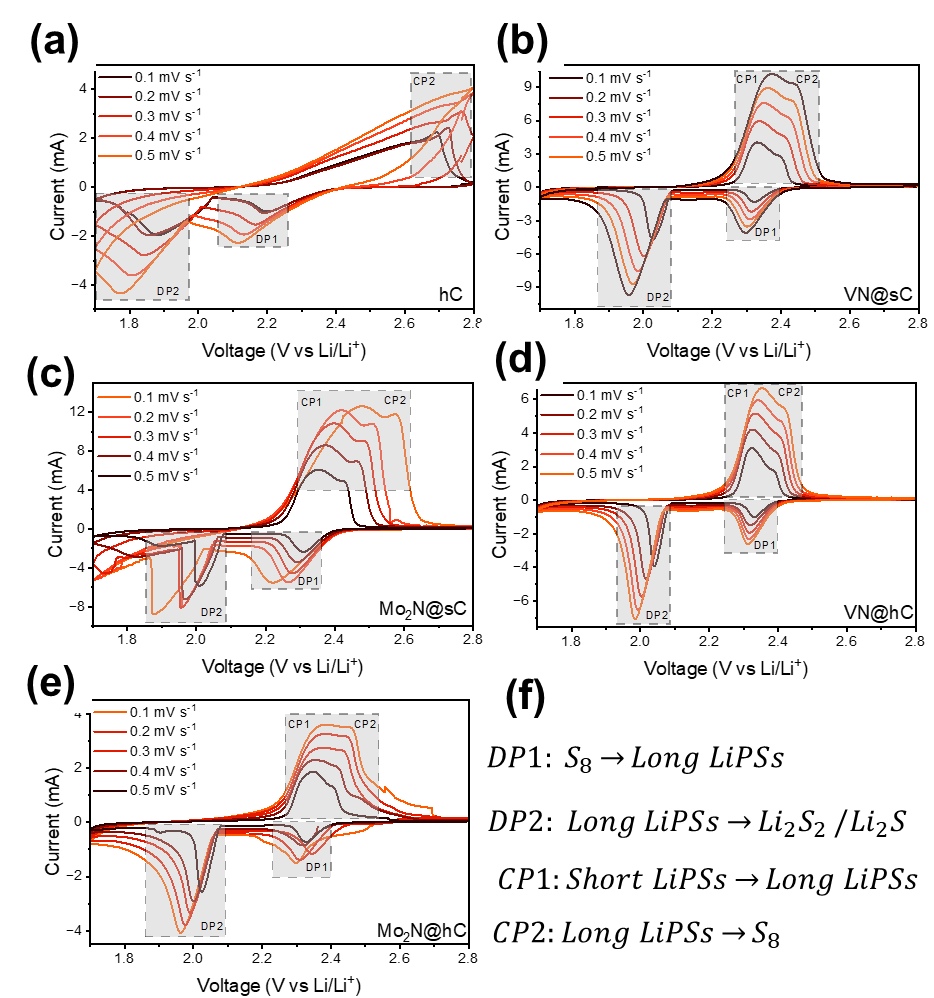


**Fig. S26**. Cyclic voltammetry (CV) curves at different scan rates (0.1-0.5 mV s^-1^). hC/S (a), VN@sC (b), Mo_2_N@sC (c), VN@hC (d), Mo_2_N@hC (e), Peaks labels (f).


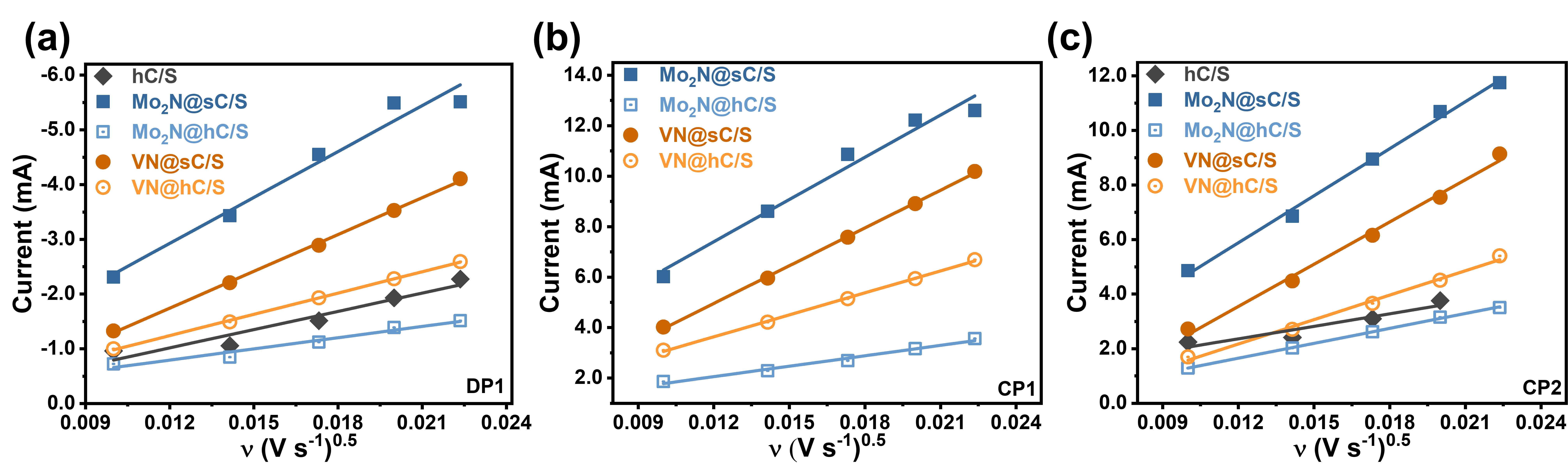


**Fig. S27.** Fitting curves for estimating D_L_ᵢ⁺. DP1 (a), CP1 (b), CP2 (c).


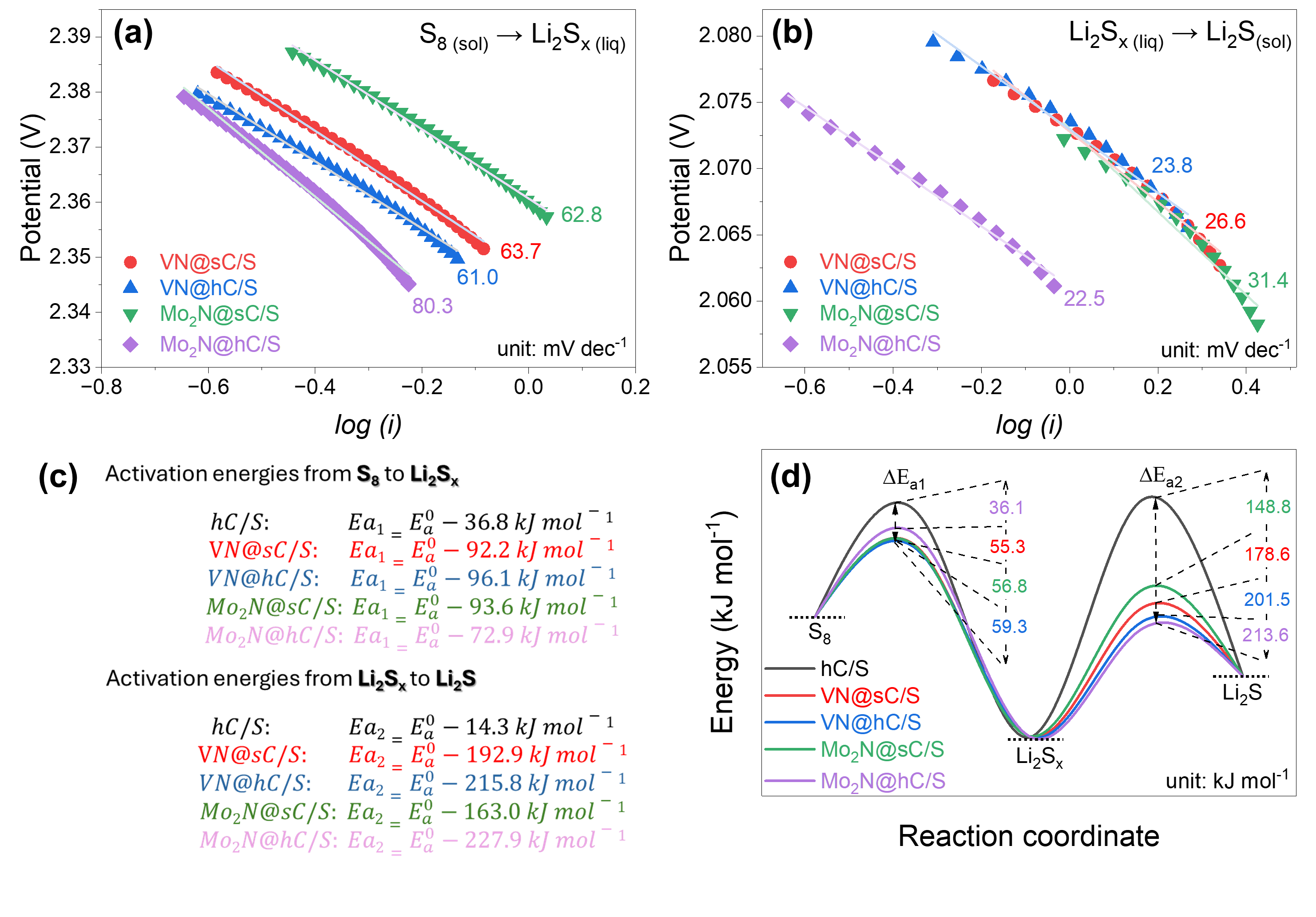


**Fig S28.** Tafel analysis of CV curves obtained at a scan rate of 0.1 mV s⁻¹. Reduction from S_8_ to Li₂Sₓ (a). Reduction from Li₂Sₓ to Li₂S (b). Summary of partially solved Tafel-derived kinetic model for all the systems (c). Difference in activation energies during the discharge process (d).

**Fig. S29**. Cyclic voltammetry (CV) curves at 3 mV s^-1^ of VN in different carbon architectures.

**Fig. S30**. Nyquist plot for TMNs embedded in hollow and solid carbon, including hC.


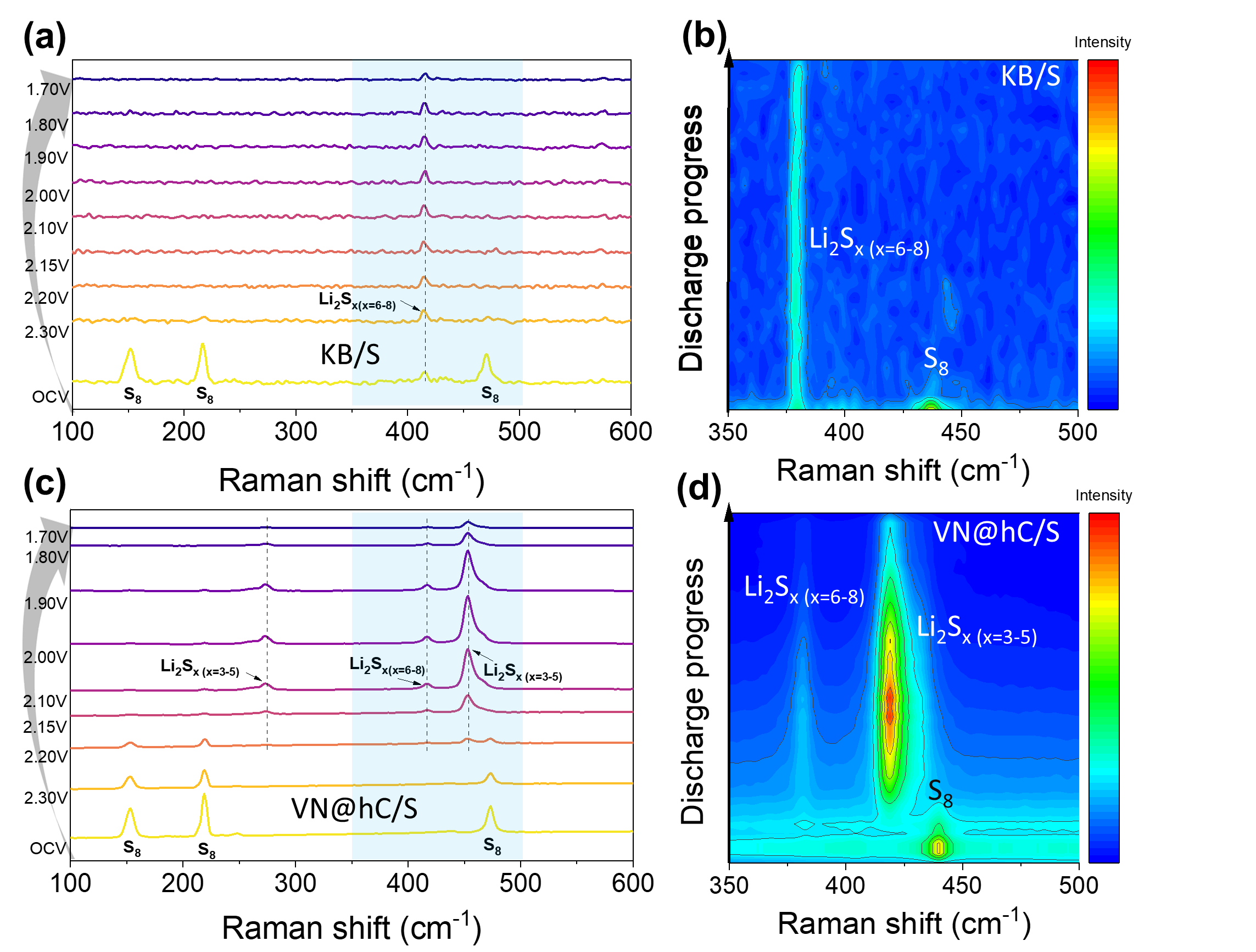


**Fig S31.** *Operando* Raman spectra of Li–S cells with a KB/S cathode at different discharge states (a), together with the corresponding heatmap of the polysulfide region (b); and a VN@hC/S cathode at different discharge states (c), with the corresponding heatmap of the polysulfide region (d). Peak assignments are comparable with prior literature. ^[S11-S12]^

**Fig. S32**. Cycling stability of TMNs embedded in hollow and solid carbon, including hC at 0.5C for 200 cycles.


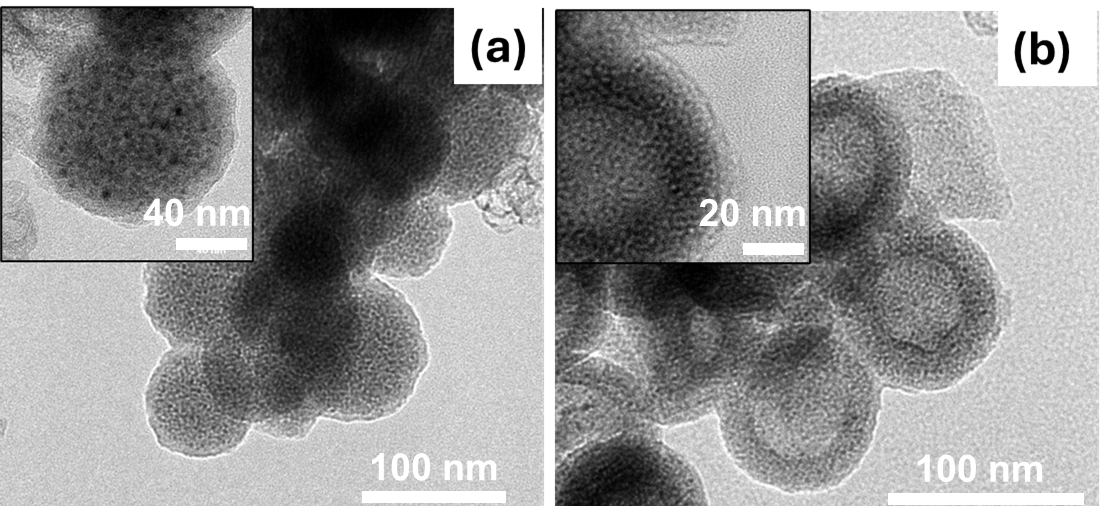


**Fig. S33**. TEM images of VN@sC after 200 cycles at 0.5C (a), and Mo_2_N@hC after 200 cycles at 0.5 C (b).

**Fig. S34**. Long-term cycling stability of VN@sC at 2C.


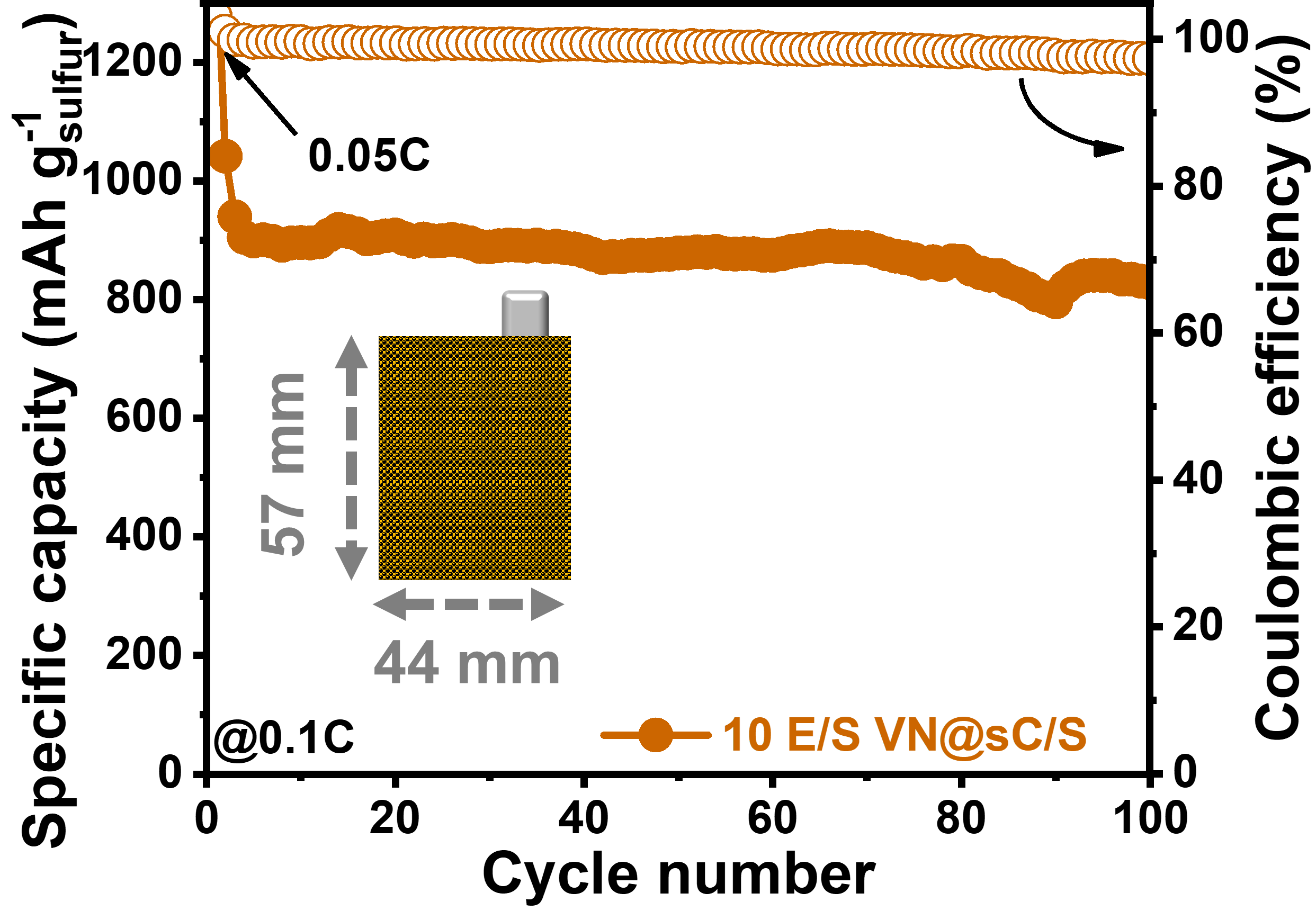


**Fig. S35**. Long-term pouch cell cycling validation of VN@sC/S (sulfur host) at 0.1C and 10 µL mg^-1^ of E/S.

**Fig. S36**. Initial charge–discharge profile of one of the twin pouch cells collected at 0.05C

**Fig. S37**. XRD pattern of VN@hC from a 110 mm x 79 mm pouch cell after 50 cycles at 0.1C.


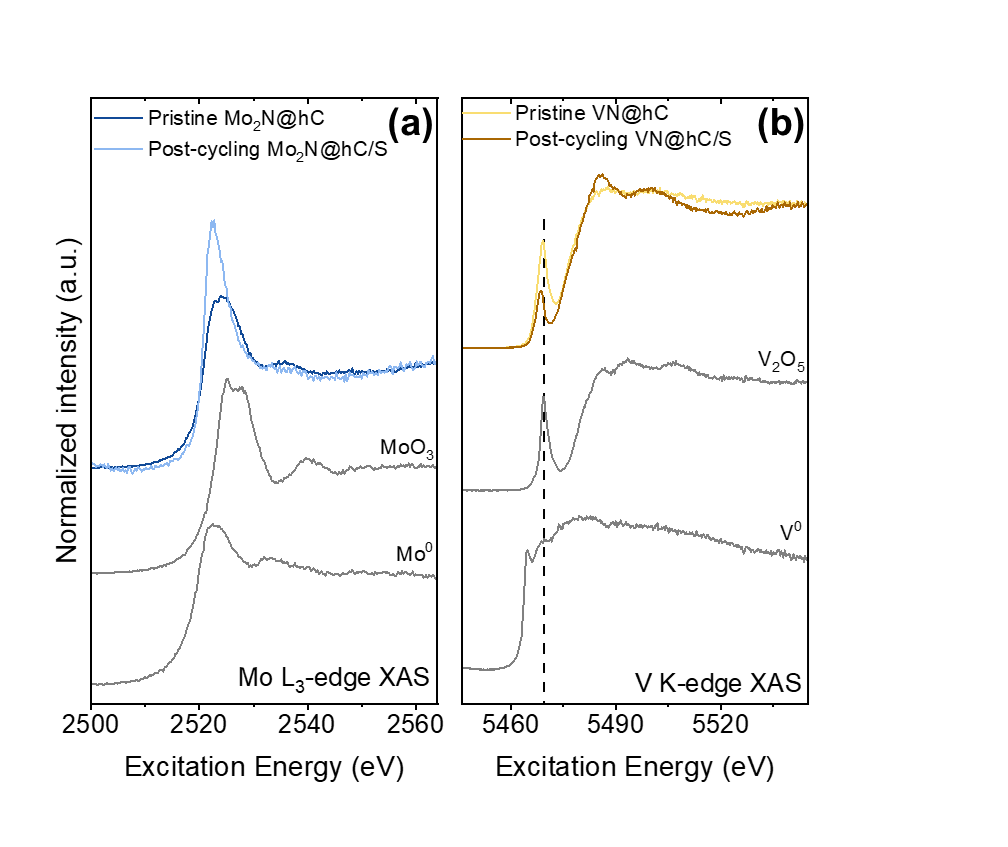


**Fig. S38**. Mo L₃-edge XAS spectra of Mo₂N@hC samples in the pristine state and after electrochemical cycling in a coin cell for 200 cycles at 0.5C, together with spectra of Mo reference compounds (a). V K-edge XAS spectra of VN@hC samples in the pristine state and after electrochemical cycling in a coin cell for 200 cycles at 0.5C, together with spectra of V reference compounds (b). Measurements were carried out in partial fluorescence yield (PFY) mode. Vertical offsets are included for clarity. A vertical dashed line is included in (b) to indicate the energy position of the pre-edge of the V_2_O_5_ spectrum.

**
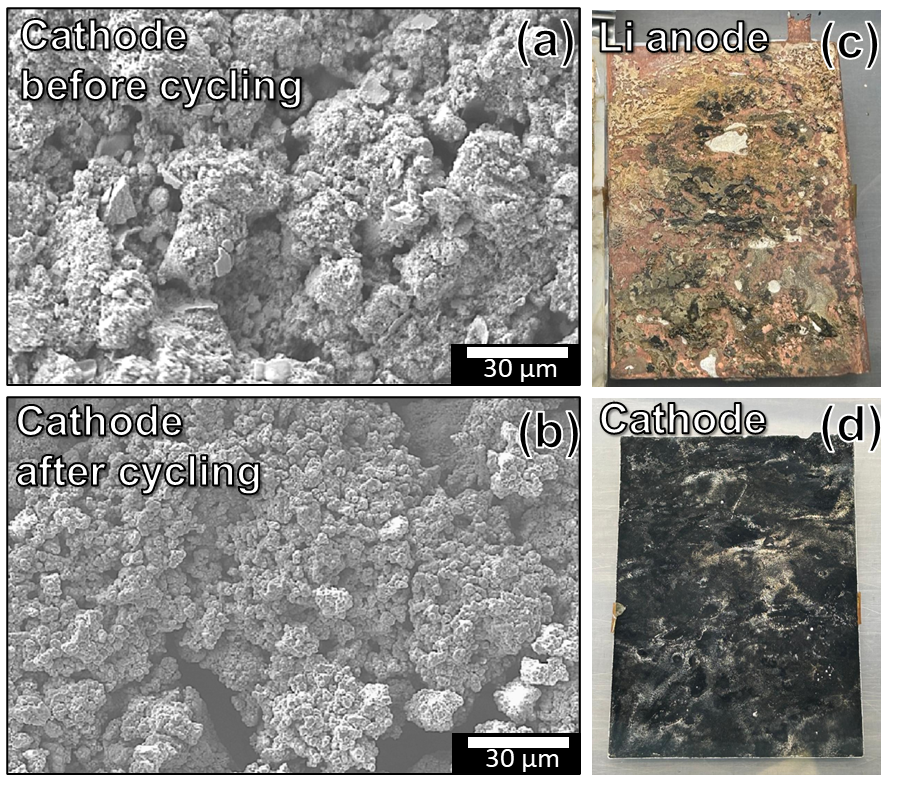
**

**Fig. S39**. SEM of VN@hC-based pouch cell with 7.9 cm x 11 cm cathode. Cathode before cycling (a). Post-cycling at 0.1 C, 50 cycles (b). Photo showing the appearance of Li anode (c), and sulfur cathode (d), after cycling.

**Table S1**. Synthesis comparison of reported TMN.

| **TMN material** | **External N-source** | **Process complexity (steps)** | **Calcination temperature (°C)** | **Hazards and sustainability** | **Calcination time (h)** | **Ref** |
| --- | --- | --- | --- | --- | --- | --- |
| **VN/G** | NH₃ | Moderate (3) | ~550 | NH₃ | 27 | [S13] |
| **VN@hC** | NH₃ | High (4) | ~480 | NH₃ + etching | 2 | [S14] |
| **VN@nativeC** | C_3_H_6_N_6_ | Moderate (3) | ~800–1000 | High temperature + solid waste | 2 | [S15] |
| **VN@hC** | HCONH_2_ | High (4) | ~900 | SiO₂ removal, corrosive chlorides | 2 | [S16] |
| **VN@N-graphene** | C_2_H_4_N_4_ | Moderate (3) | ~800 | High temperature + solid waste | 8 | [S17] |
| **Mo₂N/G** | C_2_H_4_N_4_ | Moderate (3) | ~800 | High temperature + solid waste | 5 | [S18] |
| **Mo₂N@NCNS** | HCONH_2_ | Moderate (3) | ~800 | High temperature + solid waste | 4 | [S19] |
| **Mo₂N@hC** | NH₃ | High (4) | ~800 | NH₃ | 2 | [S20] |
| **Mo₂N Mesoporous** | NH₃ | High (3) | ~800 | NH₃ + SiO₂ removal | 3 | [S21] |
| **Mo₂N@C** | CO(NH_2_)_2_ | Moderate (3) | ~680–900 | High temperature + solid waste | 2 | [S22] |
| **Self-nitrided TMNs** | **None** | **Simple (2)** | **600-700** | **NH_3_-free** | **3** | **This work** |

**Table S2**. Pore structure summary of TMNs@sC at different temperatures.

| **TMN** | **Temperature (°C)** | **Morphology** | **S_BET_^1^ (m^2^ g^-1^)** | **Pore Volume (cm^-3^ g^-1^)** |
| --- | --- | --- | --- | --- |
| **VN** | 700 | Decorated-like | 41.8 | 0.119 |
| **VN** | 800 | Decorated-like | 45.1 | 0.193 |
| **VN** | 1000 | Clusters-encapsulated | 65 | 0.245 |
| **Mo_2_N** | 600 | Decorated-like | 21.7 | 0.116 |
| **Mo_2_N** | 800 | Decorated-like | 31.6 | 0.115 |
| **Mo_2_N** | 1000 | Clusters-encapsulated | 103.5 | 0.155 |

^1^ Specific surface area from BET

**Table S3**. Elemental analysis of PILs compared to theoretical values.

| **Material** | **N (wt%)** | **C (wt%)** | **H (wt%)** |
| --- | --- | --- | --- |
| **PILs (Theoretical)** | 8.88 | 57.14 | 8.63 |
| **PILs** | 8.11 | 52.39 | 7.41 |

**Table S4**. Systematical electrochemical performance comparison of VN- and Mo₂N-based Li–S coin cells.

| **TMN material** | **Sulfur areal loading (mg cm⁻²)** | **Initial discharge capacity (mAh g⁻¹)** | **Capacity decay per cycle** | **Reference** |
| --- | --- | --- | --- | --- |
| **VN/G** | 1.9 | 1100 @ 1C | 0.1% | [S13] |
| **VN@hC** | 0.9 | 800 @ 2C | 0.02% | [S14] |
| **VN@nativeC** | 1.0 | 798 @ 1C | 0.04% | [S15] |
| **VN@hC** | 1.45 | 856.5 @ 1C | 0.05% | [S16] |
| **VN@N-graphene** | 1.2 | 1000 @ 1C | 0.11% | [S17] |
| **VN@sC** | **2.0** | **761 @ 2C** | **0.05%** | **This work** |
| **VN@hC** | **2.0** | **950 @ 1C** | **0.07%** | **This work** |
| **Mo₂N/G** | 1.8 | 1030 @ 1C | 0.05% | [S18] |
| **Mo₂N@NCNS** | 2.0 | 890 @ 2C | 0.08% | [S19] |
| **Mo₂N@hC** | 1.0 | 875 @ 0.5C | 0.24% | [S20] |
| **Mesoporous Mo₂N** | 1.1 | 990 @ 0.5C | 0.12% | [S21] |
| **Mo₂N@C** | 3.5 | 820 @ 0.5C | 0.16% | [S22] |
| **Mo₂N@sC** | **2.0** | **1067 @ 1C** | **0.1%** | **This work** |
| **Mo₂N@hC** | **2.0** | **914 @ 1C** | **0.09%** | **This work** |

**Table S5**. Electrochemical performances of representative sulfur cathodes in practical pouch cells.

| **Pouch cell system** | **Single-faced areal capacity (mAh cm⁻²)** | **Capacity retention in 50 cycles (%)** | **Metal additive in electrode (wt%)** | **Electrode size (cm^2^)** | **Sulfur content (wt%)** | **Reference** |
| --- | --- | --- | --- | --- | --- | --- |
| **CoPc@CNF** | 2.4 | 86.0 | 12.8 | 24 | 72.2 | [S23] |
| **FeP@C** | 2.95 | 95.1 | 20.0 | 26.1 | 60 | [S24] |
| **MoB** | 3.5 | 89.5 | 5.0 | 38.96 | 72 | [S25] |
| **Ti₄O₇@C** | 4.4 | 92.3 | 20.0 | 32.66 | 60 | [S26] |
| **3domsh/ZnS, CoNC** | 2.0 | 83.6 | 20.0 | 48 | 60 | [S27] |
| **G-VS₄** | 2.4 | 89.8 | 30.0 | 40 | 72 | [S28] |
| **VN₍_1−x_₎@V-NC on PP** | 3.4 | 86.35 | 4.6 | 25 | 70 | [S29] |
| **WN/Mo₂C on PP** | 1.3 | 56.5 | 24.6 | 12 | 70 | [S30] |
| **Mo₂N@hC** | **2.9** | **92.5** | **5.0** | **86.9** | **76.5** | **This work** |
| **VN@hC** | **3.5** | **83.1** | **5.0** | **86.9** | **76.5** | **This work** |

**Table S6**. The average weight of each component in a 12-layer pouch cell.

| **Material** | **Weight (g)** |
| --- | --- |
| **Sulfur** | 0.85 |
| **Double-side cathode coated on Al foil** | 1.81 |
| **Lithium coated on Cu foil** | 1.71 |
| **Electrolyte** | 3.50 |
| **Separator** | 0.54 |
| **Small components (Tabs, Tape)** | 1.11 |
| **Pouch** | 8.67 |

**Table S7** Comparison of structural characteristics, electrochemical kinetics, and Li–S battery performance of TMNs@C.

| **TMN** | **Structure** | ***S_BET_^1^* (m² g^-1^)** | **Pore size range (nm)** | **TMN content (%wt)** | **Activation energy difference*^2^* (kJ mol^-1^vs hC )** | | ***D_Li+_* *^3^***  **(cm^2^ s^-1^) x10^-7^** | **R_ct_*^4^* (Ω)** | **Best performance metrics** |
| --- | --- | --- | --- | --- | --- | --- | --- | --- | --- |
| **VN@hC** | hollow | **218.3** | 4-8 (narrow) | 39.4 | 201.5 | 2.17 | | **30** | Highest initial capacity (Large pouch cell at 0.1C, 50 cycles) |
| **VN@sC** | solid | 41.8 | 1.7-8.7 (broad) | 48.1 | 178.6 | **2.30** | | 37 | Longest term cycling (Coin cell at 2C, 650 cycles) |
| **Mo_2_N@hC** | hollow | 142.7 | **3-4 (narrow)** | 65.7 | **213.6** | 1.84 | | 36 | Stability (Large pouch cell at 0.1C, 50 cycles) |
| **Mo_2_N@sC** | solid | 21.7 | 2-9 (broad) | **67.3** | 148.8 | 2.00 | | 40 | Long term cycling (Coin cell at 1C, 700 cycles) |

^1^ Specific surface area from BET

^2^ Estimated activation energy difference for discharge peak 2 (DP2), derived from Tafel analysis of CV data collected at 0.1 mV s⁻¹.

^3^ Estimated lithium diffusion coefficients for discharge peak 2 (DP2), derived from CV data collected at 0.1 mV s⁻¹.

^4^ Charge transfer resistance

**Equation I** Randles–Sevcik equation

$$D_{{Li}^{+}}=\left( \frac{I_{p}}{2.69x10^{5}*n^{3/2}*A*C*v^{1/2}} \right)^{2}$$

where$D_{{Li}^{+}}$ represents the Li^+^ diffusion coefficient (cm^2^ s^-1^), *n* is the number of electrons transferred, *A* corresponds to area of the electrode (cm^2^), *C* corresponds to the concentration of Li^+^ (mol cm^-3^), *I_p_* is the peak current (A), and *v* corresponds to the scan rate (V s^-1^).

**Equation II** Pouch cell gravimetric energy density calculation

$$E_{d}=\frac{C*V}{m}$$

Where $E_{d}$ represents the gravimetric energy density (Wh Kg^-1^), *C* is the discharge capacity of the cell (0.96 Ah), *V* is the discharge voltage (2.1 V), *m* is the weight of the whole cell (g).

**Equation III** Tafel-based activation energy (Butler–Volmer–based)

$$E_{a}=E_{a}^{0}-\frac{RT}{b}\left. \varphi_{cathode}^{(ox|red)} \right|_{IR}$$

Where $E_{a}$is the apparent activation energy of the electrochemical reaction (kJ mol^-1^), $E_{a}^{0}$is the intrinsic activation energy (kJ mol^-1^), $R$is the gas constant (0.008314 kJ mol^-1^ K^-1^), $T$is the absolute temperature (K), $b$is the Tafel slope (mV dec^-1^) from the oxidation or reduction branch, $\phi_{\text{cathode}}\left( \text{Ox} \mid\text{Red} \right)$is the cathodic overpotential obtained from the Tafel plot, which was obtained from the middle point of the plot (mV dec^-1^).

**References**

1. D. Xie, Y. Xu, Y. Wang, X. Pan, E. Härk, Z. Kochovski, A. Eljarrat, J. Müller, C. T. Koch, J. Yuan, Y. Lu, *ACS Nano* **2022**, *16*, 10554.
2. X. Pan, Z. Kochovski, Y. L. Wang, R. M. Sarhan, E. Härk, S. Gupta, S. Stojkovikj, G. A. El-Nagar, M. T. Mayer, R. Schürmann, J. Deumer, C. Gollwitzer, J. Yuan, Y. Lu, *J. Colloid Interface Sci.* **2023**, *637*, 408.
3. J. Hafner, *J. Comput. Chem.*, **2008**, 29, 2044–2078.
4. P. E. Blöchl, *Phys. Rev. B* *1994*, 50, 17953.
5. J. P. Perdew, K. Burke, M. Ernzerhof, *Phys. Rev. Lett.* **1996**, 77, 3865.
6. S. Grimme, *J. Comput. Chem*. **2006**, 27, 1787.
7. H. J. Monkhorst and J. D. Pack. *Phys. Rev. B* **1976**, 13, 5188.
8. V. Wang, N. Xu, J.-C. Liu, et al, *Comput. Phys. Commun.* **2021**, 267, 108033.
9. M. Gorgoi, S. Svensson, F. Schäfers, G. Öhrwall, M. Mertin, P. Bressler, O. Karis, H. Siegbahn, A. Sandell, H. Rensmo, W. Eberhardt, et al, *Nucl. Instrum. Methods Phys. Res. A* **2009**, 601, 48.
10. F. Schaefers, M. Mertin, M. Gorgoi, et al, *Rev. Sci. Instrum.* **2007**, 78, 123102.
11. S. Lang, S.-H. Yu, X. Feng, M. R. Krumov, H. D. Abruña, *Nat. Commun.* **2022**, *13*, 4811.
12. W. Xu, S. Lang, K. Wang, R. Zeng, H. Li, X. Feng, M. R. Krumov, S. M. Bak, C. J. Pollock, J. Yeo, Y. Du, H. D. Abruña, *Sci. Adv.* **2023**, *9*, eadi5108.
13. Z. Sun, J. Zhang, L. Yin, G. Hu, R. Fang, H. M. Cheng, F. Li, *Nat. Commun.* **2017**, 8, 14627.
14. M. Yu, W. Fu, M. Wang, W. Wang, K. Zhu, *J. Solid State Electrochem.* **2024**, 28, 3187–3195.
15. B. Rajendran, X. Chen, Z. Li, Z. Zhan, K. B. Goh, *J. Colloid Interface Sci.* **2022**, 616, 668–678.
16. Q. Qiao, L. Ren, R. Zhang, J. Chen, M. Xu, J. Liu, H. Xu, W. Liu, Z. Chang, X. Sun, *Energy Fuels* **2021**, 35, 10219–10226.
17. E. Jing, L. Chen, S. Xu, W. Tian, D. Zhang, N. Wang, Z. Bai, X. Zhou, S. Liu, D. Duan, X. Qiu, *J. Energy Chem.* **2022**, 64, 574–582.
18. L. Qiao, L. Ren, R. Zhang, J. Chen, M. Xu, J. Liu, H. J. Xu, W. Liu, Z. Chang, X. Sun, *Energy Fuels* **2021**, 35, 10219.
19. F. Ma, Y. Chen, *Surfaces Interfaces* **2023**, 42, 103521
20. M. Cheng, Z. Xing, R. Yan, Z. Zhao, T. Ma, M. Zhou, X. Liu, S. Li, C. Cheng, InfoMat **2023**, 5, e12387.
21. G. Jiang, F. Xu, S. Yang, J. Wu, B. Wei, H. Wang, J. Power Sources **2018**, 395, 77–84.
22. L. Sun, W. Gong, J. Zhou, J. Zhang, C. Chen, X. Meng, X. Han, H. Mai, C. W. Bielawski, J. Geng, *J. Colloid Interface Sci* **2024**, 653, 1694–1703.
23. X. X. Yang, X. T. Li, C. F. Zhao, Z. H. Fu, Q. S. Zhang, C. Hu, *ACS Appl. Mater. Interfaces* **2020**, 12, 32752.
24. G. Xia, Z. Zheng, J. Ye, X. Li, M. J. Biggs, C. Hu, *Chem. Eng. J.* **2021**, 406, 126823.
25. J. He, A. Bhargav, A. Manthiram, *Adv. Mater.* **2020**, 32, 2004741.
26. S. Mei, A. Siebert, Y. Xu, T. Quan, R. Garcia-Diez, M. Bär, P. Härtel, T. Abendroth, S. Dörfler, S. Kaskel, Y. Lu, *Batter. Supercaps.* **2022**, 5, 2100398.
27. C. Zhao, G. L. Xu, Z. Yu, L. Zhang, I. Hwang, Y. X. Mo, Y. Ren, L. Cheng, C. J. Sun, Y. Ren, X. Zuo, J. T. Li, S. G. Sun, K. Amine, T. Zhao, *Nat. Nanotechnol.* **2021**, 16, 166.
28. L. Luo, J. Li, H. Yaghoobnejad Asl, A. Manthiram, *ACS Energy Lett*. **2020**, 5, 1177.
29. Y. Zhang, C. Ma, C. Zhang, L. Ma, S. Zhang, Q. Huang, C. Liang, L. Chen, L. Zhou, W. Wei, *Chem. Eng. J.* **2023**, 451, 139410.
30. Y. Wang, Y. Pu, L. Yuan, Y. Zhang, C. Liu, Q. Wang, H. Wu*, ACS* *Appl. Mater. Interfaces* **2022**, 14, 18211.
